# Clinical and Laboratory Prediction of Histopathologically Complicated Acute Appendicitis and Associated Surgical Outcomes: A Retrospective Cohort Study

**DOI:** 10.3390/jcm15208063

**Published:** 2026-10-17

**Authors:** Volkan Gökmen, Eva Kajti, Dursun Alper Yılmaz, Adem Aslan

**Affiliations:** 1 Department of Nursing, Faculty of Health Sciences, Ağrı İbrahim Çeçen University, Ağrı 04100, Türkiye; vgokmen@agri.edu.tr (V.G.); dursunyilmaz@uludag.edu.tr (D.A.Y.); 2 Department of Nursing, Faculty of Health Sciences, Atlas University, İstanbul 34408, Türkiye; 3 Department of Physiology, Faculty of Medicine, Bursa Uludağ University, Bursa 16059, Türkiye; 4 Department of General Surgery, Faculty of Medicine, Ağrı İbrahim Çeçen University, Ağrı 04100, Türkiye; aslana@agri.edu.tr

**Keywords:** acute appendicitis, complicated appendicitis, histopathology, LASSO, prediction model, postoperative complications, risk stratification

## Abstract

**Background/Objectives:** Preoperative recognition of complicated acute appendicitis remains difficult, although severity influences treatment and perioperative planning. This study examined clinical and laboratory correlates of histopathologically complicated appendicitis and associated short-term outcomes and developed a preoperative prediction model. **Methods:** This single-center retrospective cohort included 500 adults undergoing appendectomy for suspected acute appendicitis (2021–2025). The primary outcome was gangrenous or perforated appendicitis on histopathology. Fifty preoperative candidate predictors were analyzed using least absolute shrinkage and selection operator (LASSO) logistic regression with nested cross-validation, calibration and predictor-selection reproducibility assessment, comparison with the Appendicitis Inflammatory Response (AIR) and Alvarado scores, and same-center temporal evaluation (2025 cohort). **Results:** Complicated histopathology was present in 111 patients (22.2%) and was associated with longer symptom duration, physiological disturbance, peritoneal signs, and systemic inflammation. The LASSO model achieved an out-of-fold area under the curve (AUC) of 0.861 (95% confidence interval [CI], 0.828–0.892; Brier score, 0.129; calibration intercept, −0.158; slope, 0.898), compared with 0.841 for AIR (difference, 0.020; 95% CI, −0.011 to 0.054; *p* = 0.221) and 0.743 for Alvarado (difference, 0.117; 95% CI, 0.067–0.169; *p* < 0.001). In the 2025 cohort, the AUC was 0.859 (95% CI, 0.783–0.926), but calibration indicated overprediction (intercept, −0.383; slope, 0.895). Thirty-day complications occurred in 15.3% and 8.0% of patients with complicated and non-complicated histopathology, respectively. **Conclusions:** Routinely available preoperative variables provided good internally validated discrimination, but the LASSO model did not significantly outperform the simpler AIR score. Overprediction in the later same-center cohort indicates that independent multicenter validation and recalibration are required before clinical use.

## 1. Introduction

Acute appendicitis is among the most common causes of acute abdominal pain requiring emergency surgical assessment [1,2]. Management now depends not only on confirming the diagnosis but also on distinguishing uncomplicated from complicated disease. Severity influences the suitability of antibiotic-first treatment, urgency of intervention, antimicrobial duration, postoperative surveillance, and expected morbidity [3,4].

Preoperative separation of uncomplicated and complicated appendicitis remains difficult. Definitions vary according to radiological, operative, and histopathological criteria [3,5], which describe related but nonidentical aspects of disease. Reliable severity estimation is especially relevant when nonoperative treatment is considered for selected patients with uncomplicated disease [2,4].

Higher C-reactive protein (CRP), leukocytosis, neutrophilia, fever, longer symptom duration, and peritoneal signs have repeatedly been associated with gangrene or perforation [6,7,8]. Lymphopenia and increased neutrophil-to-lymphocyte ratio (NLR) may reflect greater systemic inflammatory activation, while hyponatremia has emerged as another marker of advanced intra-abdominal inflammation [9]. No single laboratory marker, however, provides adequate individual-level discrimination.

The Alvarado and Appendicitis Inflammatory Response (AIR) scores combine symptoms, signs, and laboratory findings [10,11]. They were designed primarily for diagnostic risk stratification rather than histopathological severity, and their performance varies with case mix, prevalence, diagnostic pathways, and outcome definition [6].

Computed tomography can identify appendiceal enlargement, appendicolith, periappendiceal fluid, extraluminal air, abscess, and marked fat stranding, but individual imaging signs do not consistently distinguish complicated disease across settings [5,6,12]. Imaging-dependent tools may also be limited by availability, radiation exposure, reporting variability, and local practice. Models based on rapid, routinely collected clinical and laboratory variables therefore remain attractive if they are well calibrated and validated.

Multivariable severity models increasingly combine clinical, biochemical, and imaging information. The original scoring system developed by Atema et al. [5] and the prospectively validated Scoring System of Appendicitis Severity 2.0 [13] illustrate this shift from isolated markers to individualized probabilities. Recent work has also shown that inflammation–metabolism indices can discriminate complicated or perforated appendicitis, although their incremental value over routine variables remains uncertain [14]. Models developed from many correlated predictors require penalization, strict separation of model tuning from evaluation, and calibration assessment to limit overfitting.

Severity also predicts resource use and postoperative morbidity. In a recent cohort study, conversion from laparoscopic to open appendectomy was linked to markers of operative complexity and worse postoperative outcomes [15]. More broadly, advanced appendicitis has been associated with longer hospital stay, greater antimicrobial requirements, and more postoperative complications [16,17,18].

Operative and histopathological classifications are not interchangeable. Surgeons assess contamination, peritonitis, abscess, and surrounding tissues, whereas pathology characterizes the resected appendix. Clinically relevant discrepancies have been reported [19,20]. Histopathology remains important for confirming disease, grading inflammation, and identifying unexpected abnormalities [21].

Numerous models for predicting complicated appendicitis have already been proposed, ranging from scores based on clinical and laboratory variables [6,7] to models that also incorporate imaging findings [5,13] or composite inflammatory indices [14]. Their reported performance, however, varies with case mix and outcome definition [6]. The principal gap is therefore not the absence of predictive models but the scarcity of models based on routinely available preoperative data that have been developed and evaluated using a rigorous and transparent strategy. Accordingly, the intended contribution of the present study lies less in proposing another prediction model than in its evaluation framework: penalized estimation with strict separation of model tuning from performance assessment through nested cross-validation, explicit calibration assessment, bootstrap assessment of predictor-selection reproducibility, paired comparison with established clinical scores, and same-center temporal evaluation, complemented by clinicopathological outcome analysis in one adult cohort.

The present study therefore examined associations between preoperative findings and histopathologically complicated appendicitis, developed and internally validated a penalized LASSO prediction model, compared its discrimination with the AIR and Alvarado scores, assessed temporal performance in a later same-center cohort, and evaluated associations between histopathological severity and 30-day postoperative outcomes. The model was developed specifically in adults already selected for appendectomy and was not intended to determine eligibility for nonoperative management.

## 2. Materials and Methods

### 2.1. Study Design and Population

This retrospective cohort study included adult patients who underwent appendectomy for suspected acute appendicitis at Ağrı Training and Research Hospital, Ağrı, Türkiye, between January 2021 and December 2025.

Patients were eligible if they were aged ≥18 years, underwent appendectomy because of clinical and/or radiological suspicion of acute appendicitis, and had a definitive histopathological examination of the resected appendix. The analytical cohort was defined according to the clinical setting in which a decision for appendectomy had been made rather than being retrospectively restricted to patients with histologically confirmed appendicitis. Accordingly, negative appendectomies were retained in the overall cohort and analyzed within the non-complicated group where appropriate.

A total of 500 patients fulfilled the eligibility criteria and constituted the final study cohort. The study was conducted in accordance with the Declaration of Helsinki. Ethical approval was obtained from the Ağrı İbrahim Çeçen University Scientific Research Ethics Committee on 9 July 2026 (Decision No. 459). The approval was formally communicated by the Rectorate of Ağrı İbrahim Çeçen University on 10 July 2026 (Document No. E-95531838-050.99-184225). All required institutional permissions for retrospective data collection at Ağrı Training and Research Hospital were obtained before data extraction. Because the study consisted exclusively of retrospective analysis of routinely collected clinical data, no additional intervention or study-specific procedure was performed. Personally identifiable information was excluded from the analytical dataset. The report was prepared with reference to the Strengthening the Reporting of Observational Studies in Epidemiology (STROBE) statement and TRIPOD+AI guidance for prediction-model studies [22,23]. A completed TRIPOD+AI checklist with manuscript section and page references is provided as a separate supplementary file. No prospectively registered or time-stamped statistical analysis protocol was available; accordingly, the candidate-predictor set and sensitivity analyses are not described as prespecified. Data extraction began on 20 July 2026, after ethics approval on 9 July 2026 and formal institutional authorization.

### 2.2. Study Size and Cohort Assembly

The study included all eligible records available during the fixed January 2021–December 2025 study period; therefore, the development sample size was constrained by the available retrospective cohort rather than determined prospectively. The final cohort comprised 500 patients, including 111 patients with the primary outcome, corresponding to approximately 2.2 outcome events per candidate predictor considered in the primary model. Contemporary guidance emphasizes that sample-size requirements for prediction-model development depend on the number of candidate predictor parameters, outcome prevalence, and anticipated model performance rather than on a fixed events-per-variable rule alone [24]. For illustration, applying these criteria post hoc with 50 candidate predictor parameters, an outcome prevalence of 22.2%, a target expected shrinkage factor of 0.9, and an anticipated Cox–Snell R^2^ of 0.25 (approximated from the cross-validated log-loss of the primary model) indicates a minimum of approximately 1540 patients, corresponding to approximately 340 outcome events, which is roughly three times the size of the available cohort. Accordingly, the available sample should be regarded as limited relative to the breadth of the 50-predictor candidate set. LASSO penalization and nested cross-validation were used to reduce overfitting and optimism, but these methods do not eliminate uncertainty arising from limited effective sample size. The resulting model was therefore treated as a development model requiring further external validation.

### 2.3. Data Collection

Data were retrospectively extracted from electronic medical records, emergency department documentation, laboratory databases, radiology reports, operative notes, histopathology reports, discharge records, and postoperative follow-up documentation.

Collected variables encompassed demographic characteristics, comorbidities, presenting symptoms, physical examination findings, vital signs, routine laboratory parameters, inflammatory indices, clinical scoring systems, imaging findings, operative characteristics, histopathological findings, and 30-day postoperative outcomes. For the prediction analyses, only variables documented before surgery were used. Because data were retrospectively abstracted from routine clinical records, the exact timing of individual vital-sign, physical-examination, and laboratory measurements relative to analgesia, intravenous fluid administration, or antibiotic treatment could not be reconstructed consistently for all patients. The analytical dataset therefore reflects documented preoperative clinical information rather than a prospectively standardized treatment-naïve assessment. Physical examination findings, including rebound tenderness, guarding, and rigidity, were based on the contemporaneous clinical documentation and were not reassessed centrally.

Demographic and clinical variables included age, sex, body mass index (BMI), smoking status, major comorbidities, Charlson Comorbidity Index, American Society of Anesthesiologists (ASA) physical status, previous abdominal surgery, anticoagulant or antiplatelet use, referral status, prehospital antibiotic exposure, symptom duration, pain severity assessed using a visual analogue scale (VAS), body temperature, heart rate, respiratory rate, blood pressure, peripheral oxygen saturation, migration of pain, anorexia, nausea or vomiting, right lower-quadrant tenderness, rebound tenderness, guarding, rigidity, diarrhea, constipation, and dysuria.

Laboratory parameters included white blood cell count, neutrophil percentage and absolute neutrophil count, absolute lymphocyte and monocyte counts, hemoglobin, red cell distribution width, platelet count, mean platelet volume, C-reactive protein (CRP), glucose, creatinine, serum sodium, total bilirubin, and serum albumin where available. Albumin was not included among the 50 candidate predictors in the primary LASSO model because it was not available for all patients; albumin-based descriptive analyses were therefore performed using the available-case sample. Derived inflammatory indices, including the neutrophil-to-lymphocyte ratio (NLR), platelet-to-lymphocyte ratio (PLR), monocyte-to-lymphocyte ratio (MLR), systemic immune-inflammation index (SII), and systemic inflammation response index (SIRI), were analyzed separately. CRP-to-albumin ratio was likewise evaluated descriptively among patients with available albumin measurements.

The Alvarado score and Appendicitis Inflammatory Response (AIR) score were recorded as established clinical comparators.

### 2.4. Imaging Assessment

Ultrasonography and computed tomography (CT) were performed according to routine clinical indications and the treating physician’s judgment rather than according to a study-specific imaging protocol. Recorded imaging characteristics included appendiceal diameter, wall thickness, non-compressibility, hyperemia, periappendiceal inflammatory changes, appendicolith, free fluid, fat stranding, abscess, phlegmon, extraluminal air, and the radiologist’s overall interpretation where available. Because imaging was not obtained systematically according to a study-specific protocol and its use depended on routine clinical judgment, imaging variables were not incorporated into the primary LASSO model. The study was therefore designed to evaluate an imaging-independent clinical–laboratory model and was not intended to quantify the incremental predictive value of ultrasonography or CT.

### 2.5. Operative and Histopathological Assessment

Operative variables included time to surgery, operative approach, conversion to open surgery, surgeon level, operative duration, estimated blood loss, appendiceal stump closure technique, irrigation, drain placement, gangrene, perforation, purulent intra-abdominal fluid, localized or generalized peritonitis, and intra-abdominal abscess. For analysis, the recorded intraoperative-complicated variable was treated as the operating surgeon’s overall intraoperative classification as documented in the operative record. Individual macroscopic findings, including gangrene, perforation, purulent intra-abdominal fluid, localized or generalized peritonitis, and intra-abdominal abscess, were abstracted separately. These individual findings were additionally used to construct a separate macroscopic composite outcome for sensitivity analysis.

All resected appendices underwent histopathological examination as part of routine institutional care. For the present study, histopathological classification was abstracted from the final pathology report rather than generated through a study-specific adjudication process. Gangrenous and perforated appendicitis were classified according to the final diagnostic terminology recorded in the pathology report. Information on whether individual reporting pathologists were blinded to clinical or intraoperative findings, whether a standardized pathology reporting proforma was used, and the specific microscopic criteria used to assign perforation was not consistently available in the retrospective analytical dataset; therefore, no assumptions regarding these aspects were made.

### 2.6. Primary Outcome

The primary outcome was histopathologically complicated appendicitis. Histopathologically complicated appendicitis was defined by a definitive pathological diagnosis of gangrenous or perforated appendicitis. In the analytical dataset, these diagnostic categories were mutually exclusive and comprised 54 gangrenous and 57 perforated cases, respectively. Patients with neither diagnosis were classified as having non-complicated histopathology for the primary binary analysis. Histopathological classification was selected as the primary reference outcome because it provided a specimen-based assessment recorded independently of the preoperative variables used for prediction.

### 2.7. Secondary Outcomes

Secondary outcomes included operative duration, postoperative antibiotic duration, time to oral intake, time to first flatus, length of hospital stay, any postoperative complication within 30 days, surgical-site infection, postoperative intra-abdominal abscess, postoperative ileus, percutaneous drainage, intensive care unit admission, emergency department revisit, 30-day readmission, reoperation, major postoperative morbidity, and 30-day mortality. Postoperative complications were classified according to the Clavien–Dindo classification, with grade ≥III considered major morbidity. The agreement between intraoperative and histopathological classification of complicated appendicitis was additionally evaluated. Thirty-day outcomes were ascertained from discharge records and postoperative follow-up documentation.

Any 30-day postoperative complication was defined as the occurrence of at least one documented postoperative adverse event within 30 days, including superficial or deep surgical-site infection, intra-abdominal abscess, postoperative ileus, postoperative bleeding, pneumonia, urinary tract infection, acute kidney injury, urinary retention, deep-vein thrombosis/pulmonary embolism, or death. Complications of all Clavien–Dindo grades were included in this composite, whereas grade ≥III events were additionally classified as major morbidity. Emergency department revisits and readmissions were analyzed as separate secondary outcomes and were not, by themselves, classified as postoperative complications. Individual complication categories were not mutually exclusive; therefore, a patient could contribute to more than one component outcome. Thirty-day follow-up status was available for all 500 patients in the analytical dataset and was ascertained from institutional discharge and follow-up records.

### 2.8. Descriptive and Comparative Statistical Analyses

Continuous variables were assessed for distributional characteristics using graphical methods and measures of central tendency and dispersion. Because the principal continuous variables showed non-normal or asymmetric distributions and to maintain a consistent presentation across the descriptive tables, continuous variables were summarized as median and interquartile range (IQR). For overall cohort description, age is additionally reported as mean ± standard deviation. Categorical variables were expressed as frequencies and percentages. Between-group comparisons of continuous variables were performed using the Mann–Whitney U test, whereas categorical variables were compared using Pearson’s χ^2^ test or an exact test when expected cell counts were insufficient for asymptotic inference. For Table 1, between-group effect estimates were additionally reported as differences in medians (complicated minus non-complicated) with bootstrap 95% confidence intervals based on 10,000 resamples for continuous variables and odds ratios (ORs) with 95% confidence intervals for binary variables. All statistical tests were two-sided, and *p* < 0.05 was considered statistically significant. For descriptive analyses of variables outside the primary candidate-predictor set, including imaging and other secondary variables that were not available in every patient, analyses were based on the corresponding available-case denominator. In contrast, the 50 candidate predictors used in the primary prediction model and the primary outcome were complete for all 500 patients in the analytical cohort. No missing predictor or outcome values were imputed for the primary prediction analysis.

### 2.9. Candidate Predictors for the Primary Prediction Model

The primary prediction model was restricted to demographic, clinical, physical-examination, and routine laboratory candidate variables available before surgery. Imaging variables were deliberately excluded because imaging was obtained according to routine clinical indications rather than a standardized study protocol and because the primary objective was to evaluate an imaging-independent clinical–laboratory prediction model. Operative, postoperative, and histopathological variables were excluded because they were unavailable at the intended time of prediction and their inclusion would constitute information leakage.

A total of 50 preoperative candidate predictors were evaluated: age, sex, BMI, smoking, diabetes mellitus, hypertension, coronary artery disease, chronic obstructive pulmonary disease, chronic kidney disease, immunosuppression, anticoagulant use, antiplatelet use, previous abdominal surgery, Charlson Comorbidity Index, ASA class, referral from another center, prehospital antibiotic exposure, symptom duration, pain VAS score, temperature, heart rate, respiratory rate, systolic blood pressure, diastolic blood pressure, oxygen saturation, altered mental status, pain migration, anorexia, nausea/vomiting, right lower-quadrant tenderness, rebound tenderness, guarding, rigidity, diarrhea, constipation, dysuria, white blood cell count, neutrophil percentage, absolute neutrophil count, absolute lymphocyte count, absolute monocyte count, hemoglobin, red cell distribution width, platelet count, mean platelet volume, CRP, glucose, creatinine, serum sodium, and total bilirubin.

Data completeness was assessed before model fitting. Complete availability across the analytical cohort was one of the considerations used to define the candidate set: among the preoperative clinical and laboratory variables collected, serum albumin (available in 439 patients) and the derived CRP-to-albumin ratio were not entered as candidate predictors for this reason, whereas imaging variables were excluded because imaging was not protocolized (Section 2.4). All 500 patients in the analytical cohort had recorded values for each of the 50 candidate predictors and for the primary histopathological outcome. Consequently, the primary prediction analysis was performed on all 500 patients without predictor or outcome imputation. No artificial values were assigned to replace missing measurements. Composite scores such as the AIR and Alvarado scores were not entered into the LASSO model because their component variables were already represented individually and their inclusion could introduce redundant information. Similarly, NLR, PLR, MLR, SII, and SIRI were excluded from the primary model because they are mathematically derived from hematological variables already included as individual predictors. Their incremental predictive value was evaluated separately in sensitivity analyses. ASA physical status was coded as an ordinal integer variable corresponding to the assigned class (class I = 1, class II = 2, and so on) and entered as a single linear term, both as a candidate predictor and as an adjustment covariate in the postoperative-outcome models; the corresponding odds ratios therefore represent the change associated with a one-class increase.

### 2.10. LASSO Penalized Logistic Regression

A least absolute shrinkage and selection operator (LASSO) logistic regression model was used to estimate the probability of histopathologically complicated appendicitis. LASSO applies an L1 penalty to the logistic regression coefficients, shrinking less informative coefficients toward zero and thereby reducing model complexity in the presence of numerous potentially correlated predictors. Continuous and binary predictors were standardized within each training dataset before model fitting. Importantly, scaling parameters were estimated exclusively from the corresponding training partition and subsequently applied to the validation partition, thereby preventing preprocessing-related information leakage. The strength of penalization was controlled using the inverse regularization parameter C, with smaller values corresponding to stronger L1 penalization. Penalty selection was based on cross-validated discrimination within the training data. The final full-cohort model specification corresponding to the reported coefficients used C = 0.17782794. After completion of internal validation, this final model was fitted to the complete analytical cohort solely to provide a reproducible model specification; performance estimates from this full-cohort fit were not used as validation results. The complete intercept, non-zero coefficients, and predictor scaling parameters are provided in Supplementary Table S6.

### 2.11. Nested Cross-Validation and Internal Validation

To obtain an optimism-reduced estimate of predictive performance, the LASSO model was evaluated using nested stratified cross-validation. The outer validation loop consisted of stratified 10-fold cross-validation. In each outer iteration, 90% of patients constituted the training set and the remaining 10% constituted an untouched test set. Within each outer training set, a separate stratified five-fold inner cross-validation procedure was performed to select the optimal LASSO penalty according to the area under the receiver operating characteristic curve (AUC).

AUC was used as the primary inner-loop tuning criterion because discrimination between histopathologically complicated and non-complicated appendicitis was the principal predictive objective. Because optimizing discrimination may not necessarily optimize probabilistic accuracy or calibration, a sensitivity analysis repeated the complete nested cross-validation procedure using logistic log-loss (binomial deviance) rather than AUC as the inner-loop tuning criterion. All other preprocessing, candidate predictors, cross-validation partitions, and model specifications were kept unchanged. Following penalty selection, the model was refitted using the complete outer training set and applied to the corresponding outer test fold. Consequently, each patient’s predicted probability was generated by a model that had not been trained or tuned using that patient’s data. Predictions from all outer test folds were combined to generate a complete set of out-of-fold predicted probabilities for the entire cohort. These out-of-fold probabilities formed the basis of the principal internally validated performance estimates.

Because performance estimates from a single cross-validation partition may depend on the specific allocation of patients to folds, a repeated nested cross-validation sensitivity analysis was also performed. The complete 10-fold outer and 5-fold inner cross-validation procedure was repeated 10 times using different random partitions while preserving the same candidate predictors, fold-wise preprocessing, regularization grid, and AUC-based inner-loop tuning criterion. Discrimination, Brier score, and calibration parameters were calculated separately for each repetition and summarized across repetitions using the mean, standard deviation, and range.

### 2.12. Model Performance

Model discrimination was assessed using the area under the receiver operating characteristic curve (AUC).

The 95% CI for the primary cross-validated AUC was estimated using 1000 patient-level bootstrap resamples of the pooled out-of-fold predictions. Because model fitting, preprocessing, and penalty selection were not repeated within each bootstrap resample, this interval quantifies patient-level uncertainty conditional on the out-of-fold predictions generated by the original nested cross-validation procedure and does not incorporate the full variability arising from model retraining or fold allocation. It was therefore interpreted as a conditional bootstrap confidence interval rather than a complete estimate of model-development uncertainty. Sensitivity to fold allocation was evaluated separately using repeated nested cross-validation.

Overall probabilistic accuracy was assessed using the Brier score.

Calibration was assessed using the calibration intercept, calibration slope, grouped calibration estimates, and a smoothed calibration curve based on the pooled out-of-fold predicted probabilities. Grouped observed event proportions were accompanied by 95% confidence intervals and the distribution of predicted probabilities was displayed to indicate data density across the risk range. Ideal calibration corresponds to an intercept of 0 and a slope of 1. The Brier score was additionally benchmarked against the null Brier score, calculated as π(1 − π), where π denotes the observed outcome prevalence. The scaled Brier score was calculated as 1—(Brier score/null Brier score). Because the pooled out-of-fold predictions were generated by different outer-fold models, these calibration measures were interpreted as summaries of the cross-validated prediction procedure rather than calibration of a single fixed model.

The discrimination of the LASSO model was compared with the AIR and Alvarado scores. Because predictions and scores originated from the same patients, differences in discrimination were assessed using paired patient-level resampling rather than treating the models as independent.

Sensitivity, specificity, positive predictive value, and negative predictive value were considered secondary descriptive performance measures. Threshold-based operating characteristics were treated as exploratory and were not interpreted as validated clinical decision criteria.

No fairness-specific modeling or formal subgroup performance analysis was performed. The single-center sample and number of outcome events were considered insufficient for reliable estimation of differential model performance across sociodemographic subgroups.

### 2.13. Decision Curve Analysis

As a post hoc exploratory assessment of clinical utility, decision curve analysis was performed across threshold probabilities from 0.05 to 0.50. Net benefit was calculated from the out-of-fold probabilities generated during nested cross-validation. To permit comparison on the probability scale, the AIR score was converted to predicted probabilities using logistic recalibration performed within each corresponding outer-training fold and subsequently applied to the held-out fold, thereby avoiding information leakage. The LASSO and AIR strategies were compared with treat-all and treat-none approaches. Because no universally accepted decision threshold exists for histopathologically complicated appendicitis, the decision-curve analysis was considered exploratory.

### 2.14. Bootstrap Predictor-Selection Reproducibility

Predictor-selection reproducibility was explored using 1000 patient-level bootstrap resamples. The regularization parameter was first selected in the complete analytical cohort using stratified cross-validation and was then held fixed across bootstrap resamples. For each resample, the LASSO model was refitted and the proportion of samples in which each predictor retained a non-zero coefficient was calculated. Because the penalty parameter was not reselected within each bootstrap sample, these selection frequencies may overestimate the reproducibility of predictor retention and were therefore interpreted descriptively rather than as formal measures of variable importance or selection certainty. The frequencies therefore describe the reproducibility of predictor retention at a fixed level of penalization and do not reproduce the variability of the complete model-building process, including penalty selection. No prespecified threshold was used to classify predictors as “stable.”

Because several candidate predictors were correlated, particularly hematological variables, selection frequencies were interpreted as reflecting reproducibility of retention within the multivariable penalized model rather than independent predictive importance. Continuous predictors were entered as linear terms on the log-odds scale after standardization; no nonlinear transformations or spline terms were included in the primary model. Linearity of the associations between continuous predictors and the log-odds of the outcome was not formally assessed; this is acknowledged as a limitation (Section 4.1).

### 2.15. Sensitivity Analysis of Derived Inflammatory Indices

A secondary sensitivity analysis examined whether inclusion of NLR, PLR, MLR, SII, and SIRI provided incremental discrimination beyond the raw clinical and laboratory predictors.

The extended model was evaluated using the same validation framework as the primary model. Model performance was compared principally on the basis of cross-validated AUC and Brier score.

This analysis was intended to determine whether mathematically derived systemic inflammatory indices added clinically useful predictive information beyond their underlying hematological components.

### 2.16. Alternative Outcome Sensitivity Analyses

To evaluate the dependence of predictive performance on outcome definition, the complete prediction procedure was repeated using alternative definitions of complicated appendicitis. First, the surgeon-documented overall intraoperative classification was used as a binary outcome. Second, a composite macroscopic complicated outcome was defined as the presence of at least one of the following operative findings: gangrene, perforation, purulent intra-abdominal fluid, localized peritonitis, generalized peritonitis, or intraoperative abscess. A further sensitivity analysis repeated the primary histopathology-based model after exclusion of negative appendectomies. The same 50 preoperative candidate predictors, fold-wise standardization, L1-penalized logistic regression, and nested stratified cross-validation framework were used. Discrimination and overall probabilistic accuracy were assessed using the AUC and Brier score, respectively, and AIR and Alvarado score AUCs were calculated for comparison.

### 2.17. Same-Center Temporal Evaluation

Same-center temporal evaluation was performed by developing the LASSO model using patients treated between 2021 and 2024 and evaluating it in patients treated during 2025. The two cohorts were drawn from the same institution and used the same eligibility criteria, outcome definition, candidate-predictor definitions, and data-collection framework; they differed primarily by calendar period. Within the 2021–2024 development cohort, model preprocessing and penalty selection were performed without use of 2025 observations, and the fitted development model was applied unchanged to the 2025 cohort. Temporal performance was summarized using AUC with 95% confidence intervals, Brier score, calibration intercept, and calibration slope; AIR and Alvarado AUCs were calculated for descriptive comparison. For an additional threshold-based temporal evaluation, a fixed high-sensitivity probability threshold was selected using only out-of-fold predictions from the 2021–2024 development cohort as the highest cutoff achieving at least 95% sensitivity and was then applied unchanged to the 2025 cohort. No 2025 observations were used to select or modify this threshold.

### 2.18. Association Between Severity Classification and Surgical Outcomes

Associations between appendicitis severity and postoperative outcomes were examined separately from the prediction-model analyses. To address the overlap between intraoperative and histopathological severity, an additional three-category analysis was performed using mutually exclusive groups: (1) uncomplicated by both intraoperative and histopathological assessment, (2) complicated intraoperatively but non-complicated histopathologically, and (3) complicated by both intraoperative and histopathological assessment. Continuous postoperative outcomes were summarized by group and compared using the Kruskal–Wallis test, whereas categorical outcomes were compared using Pearson’s χ^2^ or exact tests, as appropriate.

For the primary binary postoperative outcome of any 30-day complication, an expanded multivariable logistic regression model included the three-category severity variable together with age, sex, diabetes mellitus, ASA class, initial operative approach, conversion to open surgery, surgeon level, time from admission to surgery, and referral status. Because only 48 patients experienced a 30-day postoperative complication and conversion to open surgery was uncommon, this adjusted analysis was considered exploratory.

For the two-group secondary-outcome comparisons, continuous effect estimates were expressed as differences in medians with 95% bootstrap confidence intervals based on 10,000 resamples, and binary outcomes were summarized using odds ratios with 95% confidence intervals. To address multiplicity across the 17 secondary surgical and postoperative outcomes, Benjamini–Hochberg false-discovery-rate-adjusted *p* values (q values) were additionally reported. Because this was a retrospective observational study, these analyses were interpreted as associations rather than causal effects.

### 2.19. Statistical Software and Reproducibility

Analyses were performed in Python 3.12 (Python Software Foundation, Wilmington, DE, USA) using NumPy 2.3.5, SciPy 1.17.0, scikit-learn 1.8.0, statsmodels 0.14.6, and Matplotlib 3.10.8. The primary nested cross-validation used stratified 10-fold outer cross-validation with random_state = 15 and stratified five-fold inner cross-validation with random_state = 16. LASSO logistic regression was fitted using the liblinear solver with L1 penalization, max_iter = 3000, tol = 1 × 10^−6^, random_state = 15, and no class weighting (class_weight = None). Continuous and binary predictors were standardized within each training partition using StandardScaler. ROC AUC calculations used the standard rank-based implementation; observations with equal scores, where present for discrete clinical scores, were handled according to the library’s standard tie treatment. No exact ties were present among the primary LASSO out-of-fold predicted probabilities. Statistical preprocessing, model fitting, tuning, validation, and sensitivity analyses were implemented in reproducible scripts. The complete final-model specification, including centering and scaling parameters and coefficients, is provided in Supplementary Table S6.

During the preparation of this manuscript, the authors used ChatGPT (OpenAI, San Francisco, CA, USA; GPT-5.6) and Claude (Anthropic, San Francisco, CA, USA; Claude Opus 5.5) for the purposes of language and text editing. The authors have reviewed and edited the output and take full responsibility for the content of this publication.

## 3. Results

### 3.1. Study Population and Baseline Characteristics

**Figure 1:**
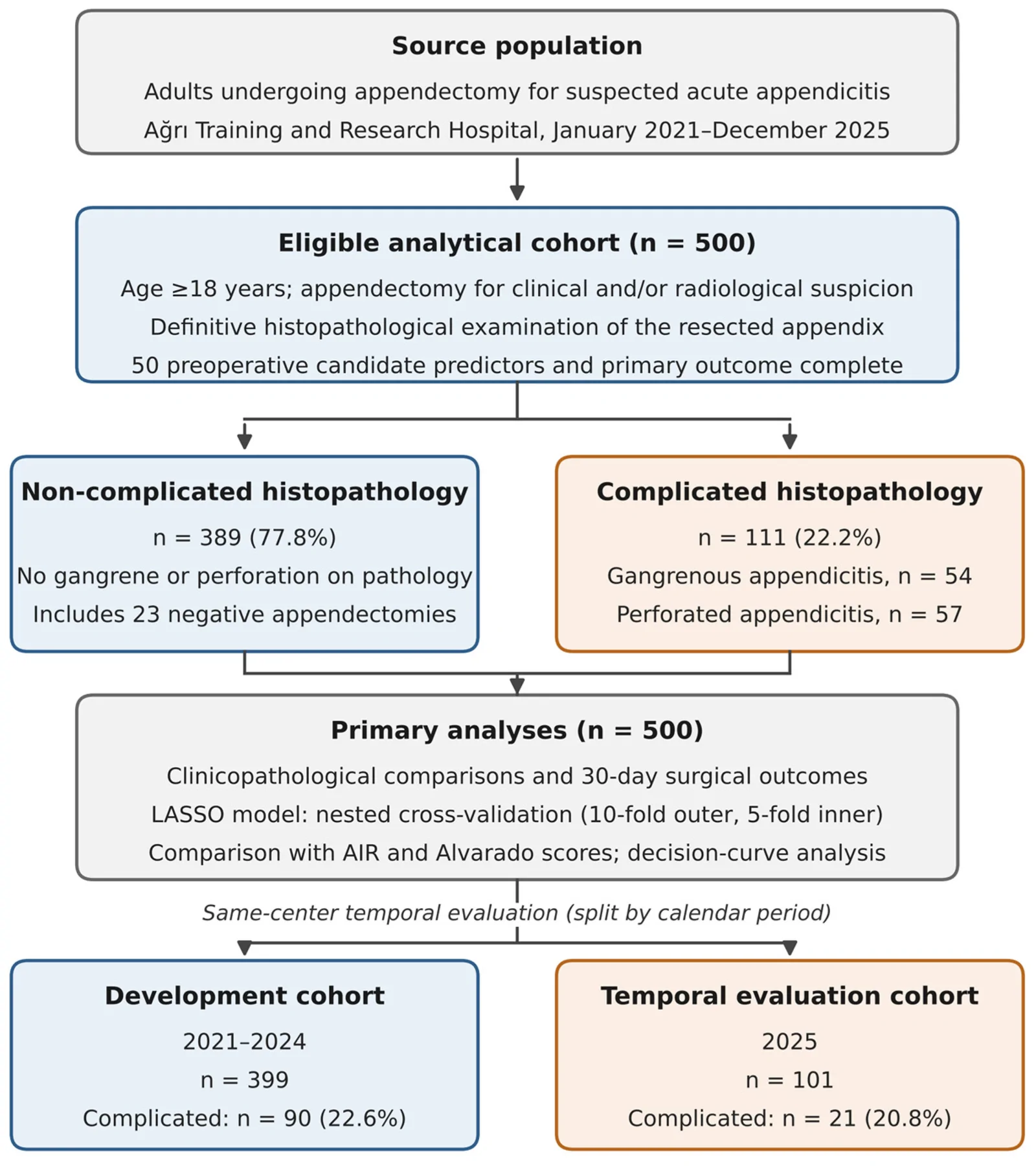
Study flow and cohort allocation. The analytical cohort included 500 adults who underwent appendectomy for suspected acute appendicitis between January 2021 and December 2025. Patients were classified according to definitive histopathology as non-complicated or complicated appendicitis. The primary analyses included clinicopathological comparisons, surgical outcome analyses, and development of a nested cross-validated LASSO prediction model. For same-center temporal evaluation, the dataset was divided into a 2021–2024 development cohort and a 2025 temporal evaluation cohort.

A total of 500 adult patients who underwent appendectomy for suspected acute appendicitis between 2021 and 2025 were included in the study. The mean age of the overall cohort was 37.5 ± 15.1 years, and 276 patients (55.2%) were male. Histopathologically complicated appendicitis was identified in 111 patients (22.2%), whereas 389 patients (77.8%) were classified as having non-complicated histopathology. Intraoperatively complicated appendicitis was documented in 192 patients (38.4%). Intraoperative gangrene and perforation were documented in 83 (16.6%) and 72 (14.4%) patients, respectively. These findings overlapped: 65 patients had gangrene without perforation, 54 had perforation without gangrene, and 18 had both findings. On definitive histopathology, 54 patients were classified as having gangrenous appendicitis and 57 as having perforated appendicitis, together constituting the 111 patients with the primary histopathologically complicated outcome. Twenty-three patients (4.6%) had a negative appendectomy. Participant flow and cohort allocation are shown in Figure 1.

Age, sex, body mass index (BMI), hypertension, smoking status, previous abdominal surgery, referral status, and prehospital antibiotic use did not differ significantly between patients with complicated and non-complicated histopathology. Diabetes mellitus was more frequent in the complicated group (15.3% vs. 7.5%, *p* = 0.011).

Patients with histopathologically complicated appendicitis had a substantially longer duration of symptoms than those with non-complicated disease [36.0 (25.6–56.0) vs. 20.3 (13.7–30.8) h, *p* < 0.001]. They also had higher pain scores, body temperature, heart rate, respiratory rate, and shock index (all *p* < 0.001). Signs of peritoneal irritation were markedly more frequent in the complicated group, including rebound tenderness, guarding, and rigidity (all *p* < 0.001). Baseline demographic and clinical characteristics according to histopathological severity are summarized in Table 1.

### 3.2. Laboratory Findings and Systemic Inflammatory Profile

Patients with histopathologically complicated appendicitis exhibited a more pronounced systemic inflammatory response. Median white blood cell count was significantly higher in the complicated group [16.21 (13.74–17.78) vs. 13.34 (11.23–15.17) × 10^3^/µL, *p* < 0.001]. Neutrophil percentage and absolute neutrophil count were likewise higher, whereas absolute lymphocyte count was markedly lower in complicated disease (all *p* < 0.001).

CRP showed a substantial between-group difference, with a median concentration of 79.2 (46.7–129.1) mg/L in patients with complicated appendicitis compared with 29.1 (14.5–56.1) mg/L in those with non-complicated disease (*p* < 0.001). Complicated appendicitis was also associated with higher serum glucose and total bilirubin concentrations and lower serum sodium levels. Among patients with available albumin measurements, serum albumin was also lower in the complicated group. All evaluated derived inflammatory indices, including NLR, PLR, MLR, SII, and SIRI, were significantly higher in the complicated group.

Both conventional clinical scores also differed substantially between groups. The median Alvarado score was 9 (8–9) in complicated appendicitis compared with 7 (6–8) in non-complicated appendicitis, while the corresponding AIR scores were 8 (8–9) and 6 (5–7), respectively (both *p* < 0.001). Detailed laboratory findings, inflammatory indices, and clinical scores are presented in Supplementary Table S1.

### 3.3. Agreement Between Intraoperative and Histopathological Severity

Intraoperatively complicated appendicitis was documented in 192 of 500 patients (38.4%), whereas 111 patients (22.2%) met the histopathological definition of complicated appendicitis. All 111 patients with histopathologically complicated appendicitis had also been classified as complicated intraoperatively. Among the 389 patients without histopathologically complicated disease, 81 were classified as complicated intraoperatively, while 308 were classified as non-complicated by both approaches.

Accordingly, the histopathologically complicated group was entirely nested within the broader surgeon-documented intraoperative classification rather than representing an independent competing classification. The overall intraoperative classification was broader than the presence of gangrene or perforation alone and incorporated the surgeon’s global assessment of macroscopic disease severity. Therefore, the 192 patients classified as complicated intraoperatively should not be interpreted as corresponding directly to the union of the separately recorded intraoperative gangrene and perforation findings.

These findings demonstrate substantial overlap, but incomplete equivalence, between intraoperative and histopathological severity classifications. The two definitions were therefore treated as related but distinct measures of disease severity, and agreement statistics were interpreted descriptively rather than as evidence that either classification represented a definitive criterion standard.

### 3.4. Association Between Severity Classification and Surgical Outcomes

Histopathologically complicated appendicitis was associated with less favorable perioperative and postoperative outcomes in the primary two-group analysis. Any postoperative complication within 30 days occurred in 17 of 111 patients (15.3%) with complicated histopathology compared with 31 of 389 patients (8.0%) with non-complicated histopathology, corresponding to an unadjusted odds ratio of 2.09 (95% CI, 1.11–3.94; *p* = 0.020).

Because intraoperative and histopathological severity classifications overlapped, an additional three-category analysis was performed. Of the 500 patients, 308 were classified as uncomplicated by both intraoperative and histopathological assessment, 81 were classified as complicated intraoperatively but non-complicated histopathologically, and 111 were classified as complicated by both assessments. Any 30-day postoperative complication occurred in 20/308 (6.5%), 11/81 (13.6%), and 17/111 (15.3%) patients, respectively (global *p* = 0.011).

In the expanded multivariable logistic regression model, compared with patients classified as uncomplicated by both assessments, the adjusted odds ratio for any 30-day complication was 1.83 (95% CI, 0.80–4.21; *p* = 0.155) for patients with intraoperative-only complicated disease and 2.53 (95% CI, 1.19–5.37; *p* = 0.016) for patients classified as complicated by both assessments. The direct comparison between the both-complicated and intraoperative-only groups was not statistically significant (adjusted OR, 1.38; 95% CI, 0.59–3.24; *p* = 0.459).

These findings indicate that postoperative complication risk increased across severity categories but do not demonstrate a clear additional association of histopathological classification beyond intraoperative severity alone. Management-dependent outcomes, including operative duration, postoperative antibiotic duration, return of gastrointestinal function, and length of stay, were therefore interpreted cautiously because they may reflect intraoperative disease assessment and local treatment protocols. Detailed two-group outcomes are presented in Table 2, and the three-category analysis is provided in Supplementary Table S8.

### 3.5. Development and Internal Validation of the LASSO Prediction Model

**Figure 2:**
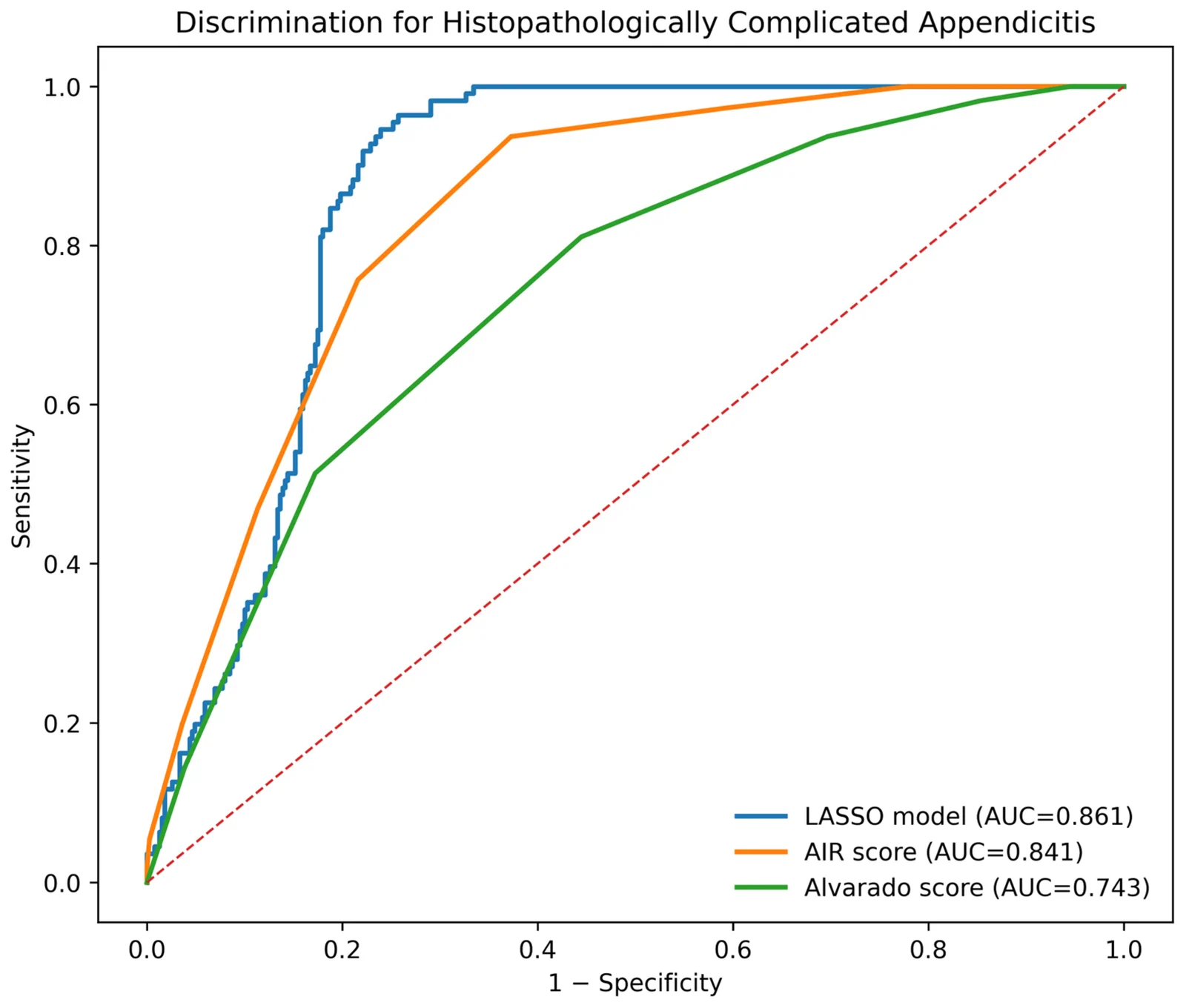
Receiver operating characteristic curves for prediction of histopathologically complicated appendicitis. The internally validated LASSO model achieved an AUC of 0.861, compared with 0.841 for the Appendicitis Inflammatory Response (AIR) score and 0.743 for the Alvarado score. LASSO, least absolute shrinkage and selection operator; AUC, area under the receiver operating characteristic curve. The dashed diagonal line represents the reference line of no discrimination (AUC = 0.5).

The resulting out-of-fold predictions demonstrated good discrimination for histopathologically complicated appendicitis, with an internally validated AUC of 0.861 (conditional bootstrap 95% CI, 0.828–0.892). For comparison, the AIR score achieved an AUC of 0.841, whereas the Alvarado score yielded an AUC of 0.743. The AUC difference between the LASSO model and AIR score was 0.020 (95% CI −0.011 to 0.054; *p* = 0.221), whereas the difference versus the Alvarado score was 0.117 (95% CI 0.067–0.169; *p* < 0.001). The predictive performance metrics are summarized in Table 3, and the corresponding receiver operating characteristic curves are shown in Figure 2.

Inspection of the underlying out-of-fold predictions showed that the visually steep portion of the LASSO ROC curve did not represent a single discontinuity or a cluster of exactly tied predicted probabilities. Between false-positive rates of approximately 0.14 and 0.20, sensitivity increased from approximately 0.53 to 0.86 through multiple closely spaced empirical ROC steps. The largest individual vertical step increased sensitivity from 0.604 to 0.667 at a false-positive rate of approximately 0.159. Thus, the apparent steep segment primarily reflects the empirical ranking of a finite number of events rather than an isolated probability tie.

**Figure 3:**
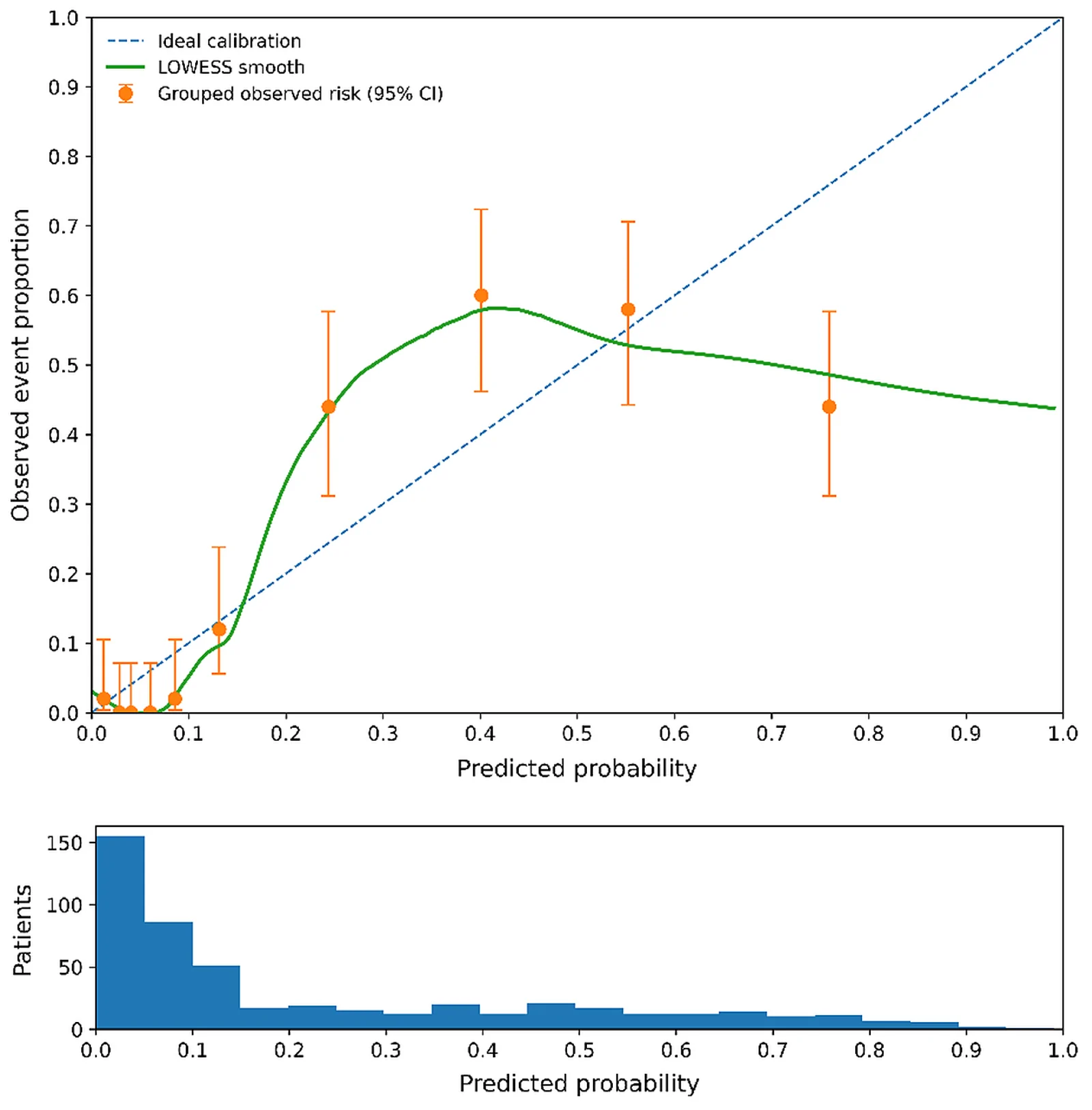
Calibration of the LASSO model for histopathologically complicated appendicitis based on pooled out-of-fold predictions from nested cross-validation. Circles represent observed event proportions within ten equal-sized groups of predicted risk (*n* = 50 per group), formed by ranking patients according to their out-of-fold predicted probability; each circle is plotted at the mean predicted probability of its group, with vertical bars indicating 95% confidence intervals. The solid curve represents a LOWESS-smoothed calibration estimate, and the dashed diagonal line indicates perfect calibration. The lower panel shows the distribution of out-of-fold predicted probabilities.

The Brier score was 0.129, compared with a null Brier score of 0.173, corresponding to a scaled Brier score of 0.253. Using a logistic recalibration model fitted to the pooled out-of-fold predictions, the calibration intercept was −0.158 (95% CI, −0.445 to 0.129) and the calibration slope was 0.898 (95% CI, 0.719–1.078). Although the overall calibration parameters were reasonably close to their ideal values, calibration was not uniform across the predicted-risk range. The grouped calibration estimates and LOWESS curve showed underestimation in parts of the intermediate-risk range and overprediction among patients with the highest predicted probabilities (Figure 3). As a sensitivity analysis addressing the choice of inner-loop optimization criterion, the complete nested cross-validation procedure was repeated using logistic log-loss rather than AUC for penalty selection, while keeping the candidate predictors, preprocessing steps, cross-validation partitions, and candidate regularization values unchanged. Performance was very similar: the log-loss-tuned model yielded an out-of-fold AUC of 0.863, a Brier score of 0.128, and a log-loss of 0.381. The corresponding calibration intercept was −0.123 and the calibration slope was 0.950, compared with −0.158 and 0.898, respectively, for the primary AUC-tuned model. Thus, discrimination and overall probabilistic accuracy were minimally affected by the tuning criterion, while calibration was slightly closer to the ideal intercept of 0 and slope of 1 with log-loss-based tuning. Detailed results are provided in Supplementary Table S9.

Performance was also evaluated across 10 repetitions of the complete nested cross-validation procedure to assess sensitivity to fold allocation. Across repetitions, the mean out-of-fold AUC was 0.856 (SD, 0.007; range, 0.844–0.864), and the mean Brier score was 0.130 (SD, 0.002; range, 0.127–0.133). The mean calibration intercept was −0.145 (SD, 0.059), and the mean calibration slope was 1.030 (SD, 0.092). These findings indicate modest variation across cross-validation partitions but broadly similar discrimination and overall probabilistic accuracy. Detailed repeated nested cross-validation results are provided in Supplementary Table S10.

In the sensitivity analysis, adding the derived inflammatory indices NLR, PLR, MLR, SII, and SIRI to the raw clinical–laboratory predictor set did not materially improve discrimination (AUC 0.865 vs. 0.861; ΔAUC 0.004, 95% CI −0.003 to 0.013; *p* = 0.302) and produced a similar Brier score (0.128 vs. 0.129). Detailed results are provided in Supplementary Table S2.

For reproducibility, a final LASSO model was subsequently fitted to the complete analytical cohort after completion of the internal-validation procedure. The final specification retained 30 predictors with non-zero coefficients and had a standardized intercept of −1.781374. The complete model specification, including predictor centering and scaling parameters, standardized coefficients, and equivalent raw-scale coefficients, is provided in Supplementary Table S6. Because this final model was fitted using the complete cohort, its apparent performance was not used as an estimate of validated model performance.

### 3.6. Bootstrap Reproducibility of Predictor Selection

**Figure 4:**
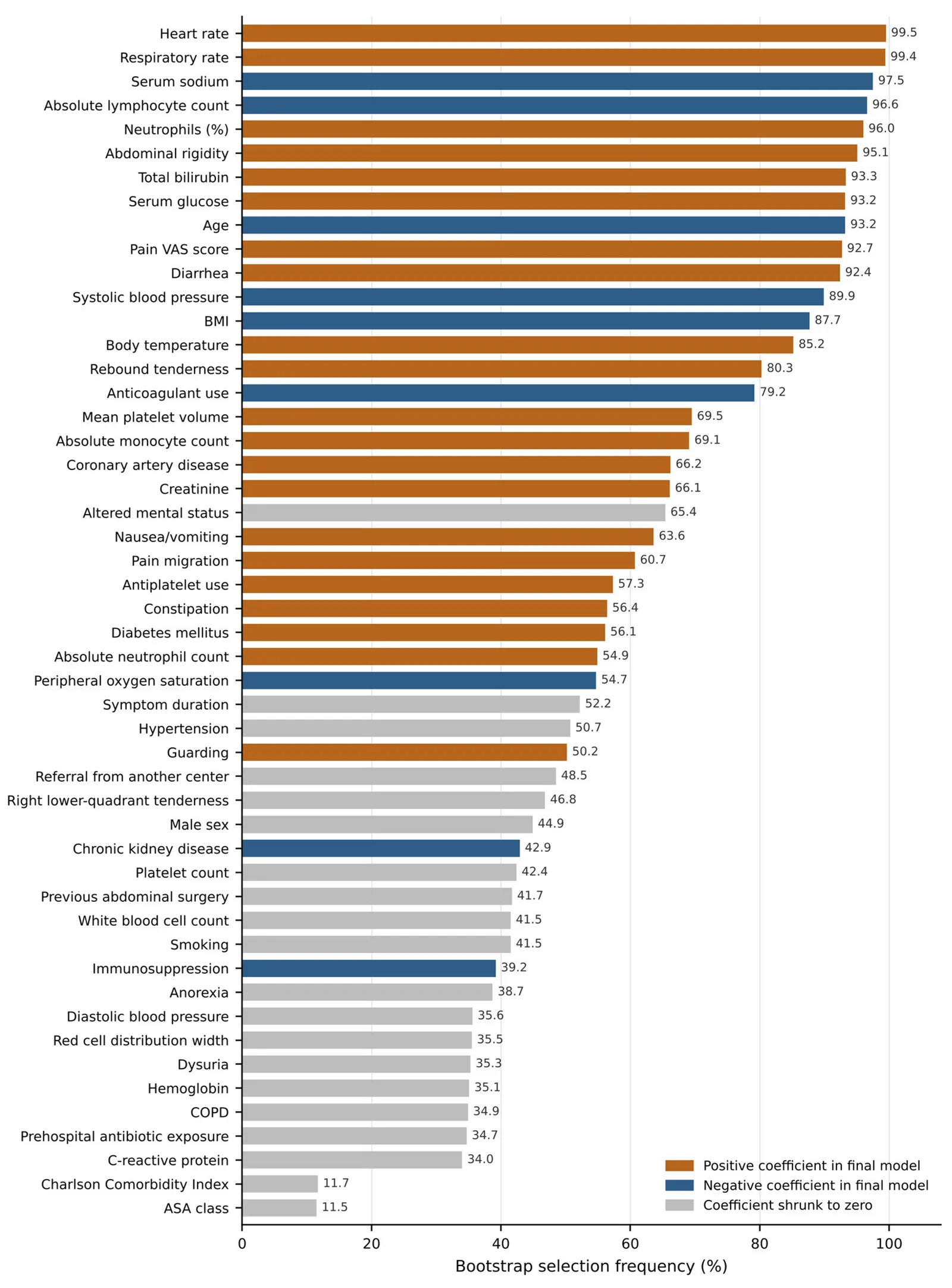
Descriptive bootstrap selection frequencies for all 50 LASSO candidate predictors. Bars indicate the percentage of 1000 bootstrap samples in which each predictor retained a non-zero coefficient using the fixed penalty selected in the complete analytical cohort. Frequencies are presented as descriptive measures of retention reproducibility and should not be interpreted as rankings of predictor importance. Bar shading indicates the sign of the corresponding coefficient in the final full-cohort model (positive, negative, or shrunk to zero; Supplementary Tables S3 and S6).

Bootstrap analysis showed that several predictors were frequently retained when the LASSO model was refitted using the fixed penalty selected in the complete cohort. Heart rate and respiratory rate had the highest selection frequencies (99.5% and 99.4%, respectively), followed by serum sodium (97.5%), absolute lymphocyte count (96.6%), neutrophil percentage (96.0%), abdominal rigidity (95.1%), total bilirubin (93.3%), age (93.2%), serum glucose (93.2%), pain VAS score (92.7%), diarrhea (92.4%), systolic blood pressure (89.9%), BMI (87.7%), body temperature (85.2%), and rebound tenderness (80.3%). In contrast, symptom duration and CRP were retained in 52.2% and 34.0% of bootstrap samples, respectively. Because the penalty was held fixed across bootstrap samples and several predictors were correlated, these frequencies should be interpreted as descriptive measures of retention reproducibility rather than rankings of predictor importance. The frequently retained predictors are summarized in Table 4, together with their standardized coefficients and direction of association in the final full-cohort model, whereas Figure 4 displays the selection frequencies of all 50 candidate predictors and complete numerical results are provided in Supplementary Table S3.

### 3.7. Decision Curve Analysis

**Figure 5:**
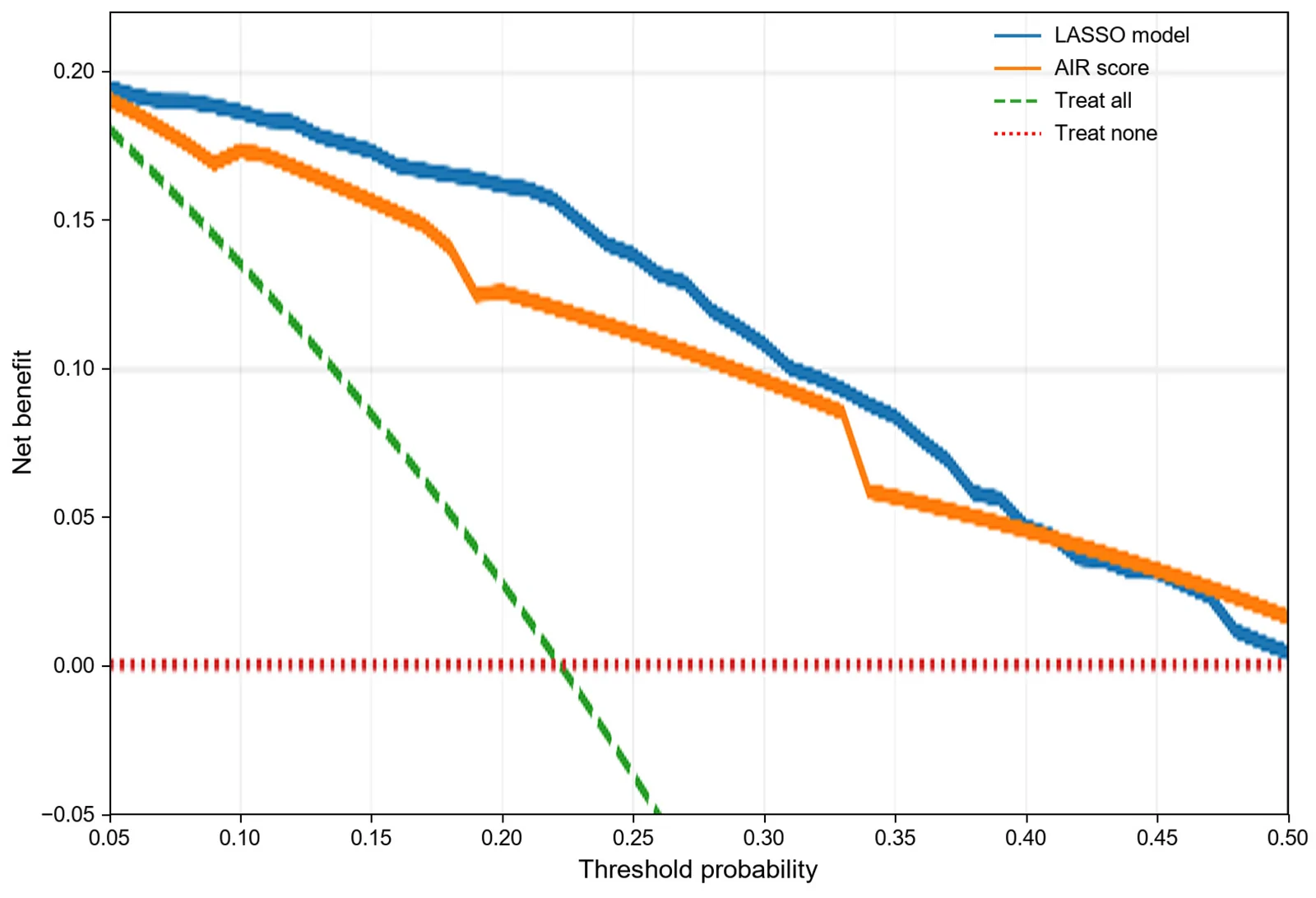
Decision curve analysis of the LASSO model and AIR score for prediction of histopathologically complicated appendicitis. Net benefit is shown across threshold probabilities from 0.05 to 0.50. The LASSO model and AIR score are compared with treat-all and treat-none strategies. AIR probabilities were generated using fold-wise logistic recalibration within the cross-validation framework. The vertical axis is truncated at −0.05 to display the clinically relevant range; the treat-all curve continues below this limit at higher thresholds.

Decision curve analysis showed that the LASSO model provided a higher net benefit than the AIR score and the treat-all and treat-none strategies across most threshold probabilities between approximately 0.05 and 0.41 (Figure 5). The incremental benefit was most evident within the 0.15–0.25 threshold range. At a threshold probability of 0.20, net benefit was 0.162 for the LASSO model and 0.126 for AIR, corresponding to a difference of 0.036 (bootstrap 95% CI, 0.016–0.058). The advantage diminished at higher threshold probabilities and was not maintained above approximately 0.42.

### 3.8. Same-Center Temporal Evaluation

Same-center temporal evaluation was performed by developing the LASSO model using patients treated between 2021 and 2024 and evaluating it in patients treated during 2025. The development cohort included 399 patients, including 90 patients (22.6%) with histopathologically complicated appendicitis, whereas the later 2025 cohort comprised 101 patients, including 21 patients (20.8%) with the primary outcome. Both cohorts were drawn from the same institution and used the same eligibility criteria, outcome definition, predictor definitions, and data-collection framework. No observations from 2025 were used for scaling, penalty selection, predictor estimation, model fitting, or threshold selection.

In the 2025 cohort, the LASSO model achieved an AUC of 0.859 (95% CI, 0.783–0.926) and a Brier score of 0.131. The AIR score yielded an AUC of 0.872 (95% CI, 0.786–0.942), whereas the Alvarado score yielded an AUC of 0.760 (95% CI, 0.637–0.871). The null Brier score was 0.165, corresponding to a scaled Brier score of 0.205.

Temporal calibration was less favorable, with a calibration intercept of −0.383 and a calibration slope of 0.895. The negative intercept indicates systematic overprediction of the absolute risk of histopathologically complicated appendicitis in the 2025 cohort, while the slope below 1 suggests that predicted risks were somewhat too extreme. Given that the temporal cohort contained only 21 outcome events, these calibration estimates are imprecise and should be interpreted cautiously.

A fixed probability threshold of 0.176 was selected using only out-of-fold predictions from the 2021–2024 development cohort, where it achieved 95.6% sensitivity and 73.1% specificity. When applied unchanged to the 2025 cohort, sensitivity was 90.5% (19/21), specificity was 73.8% (59/80), positive predictive value was 47.5%, and negative predictive value was 96.7%. Two of the 21 patients with complicated histopathology fell below this threshold. The reduction in sensitivity in the later cohort reinforces that this exploratory threshold should not be interpreted as a validated clinical rule-out criterion.

These findings represent a same-center temporal assessment rather than geographic external validation. Although discrimination was maintained in the later cohort, calibration drift was evident, and the limited number of events precludes strong conclusions regarding temporal transportability. Detailed temporal evaluation results are provided in Supplementary Table S4, and the corresponding ROC curve is shown in Supplementary Figure S1.

### 3.9. Alternative Outcome Sensitivity Analyses

Predictive performance varied according to the definition of complicated appendicitis. When the surgeon-documented intraoperative classification was used as the outcome, 192 of 500 patients (38.4%) were classified as complicated, and the LASSO model yielded an out-of-fold AUC of 0.998 (95% CI, 0.997–1.000) and a Brier score of 0.016. The corresponding AUCs for the AIR and Alvarado scores were 0.947 and 0.797, respectively.

A separate macroscopic composite based on the presence of gangrene, perforation, purulent intra-abdominal fluid, localized or generalized peritonitis, or intraoperative abscess classified 222 of 500 patients (44.4%) as complicated. For this outcome, the LASSO model yielded an AUC of 0.885 (95% CI, 0.851–0.917) and a Brier score of 0.114; the corresponding AIR and Alvarado AUCs were 0.858 and 0.735.

After exclusion of the 23 negative appendectomies, 477 patients remained, including all 111 histopathologically complicated cases. The histopathology-based LASSO model yielded an AUC of 0.840 (95% CI, 0.802–0.880) and a Brier score of 0.135, compared with AUCs of 0.833 for AIR and 0.734 for Alvarado. Thus, exclusion of negative appendectomies reduced LASSO discrimination from 0.861 to 0.840, indicating that negative appendectomies contributed modestly to discrimination but did not account for the overall predictive signal. Detailed results are provided in Supplementary Table S7.

## 4. Discussion

In this adult appendectomy cohort, complicated histopathology was accompanied by longer symptom duration, greater physiological disturbance, peritoneal signs, leukocytosis, neutrophilia, lymphopenia, higher CRP and bilirubin, and lower sodium. It was also associated with longer operations, prolonged recovery and hospitalization, and more 30-day complications. A LASSO model based only on preoperative clinical and laboratory data showed good internal and temporal discrimination. Operative and histopathological classifications overlapped but were not equivalent.

The clinical pattern agrees with earlier work linking complicated appendicitis to delayed presentation, fever, guarding, rebound tenderness, leukocytosis, and CRP [1,7,8,25]. Ben Ismail et al. reported an AIR AUC of 0.841 (95% CI, 0.801–0.882) for complicated appendicitis [25], which was similar to the AIR performance observed in the present cohort. An internally validated adult cohort that also incorporated CT findings identified guarding, rebound tenderness, CRP, appendiceal diameter, free fluid, and appendicolith as important predictors [26]. In the present study, imaging was clinician-directed rather than protocolized, and a formal comparison between clinical–laboratory and imaging-inclusive prediction strategies was therefore not attempted. Consequently, the present findings should not be interpreted as demonstrating equivalence or superiority to CT-based or CT-inclusive approaches.

Symptom duration may indicate disease progression and delays in the clinical pathway. It was substantially longer in complicated cases, consistent with prior reports [7,27]. Appendicitis is nevertheless biologically heterogeneous; progression is not uniformly linear, so duration should be interpreted as one element of a broader risk profile [1,3].

CRP was a strong univariable discriminator, as in previous studies [7,8,28]. Its lower selection frequency in the penalized multivariable model does not imply a lack of clinical value. Correlated markers compete under LASSO penalization; CRP may add little information after neutrophil predominance, lymphopenia, temperature, heart rate, and other inflammatory signals are included. Consistent with this, the CRP coefficient was shrunk to zero in the final full-cohort model (Supplementary Table S3). Bootstrap selection frequency should not be equated with the strength of an univariable association or with causal importance. For example, age showed little univariable separation between histopathological groups but was frequently retained in the multivariable penalized model. This may reflect conditional associations arising after adjustment for correlated covariates rather than a strong marginal relationship. Conversely, CRP and symptom duration showed marked univariable differences but lower bootstrap retention frequencies (34.0% and 52.2%, respectively), consistent with redistribution of predictive information among correlated clinical and inflammatory variables under L1 penalization. These findings therefore illustrate the instability of attributing individual-variable importance within a correlated predictor set.

Complicated cases had higher neutrophil counts, lower lymphocyte counts, and higher NLR, systemic immune-inflammation index, and systemic inflammation response index. These patterns agree with contemporary laboratory studies [8,29] and with recent evidence on combined inflammatory-metabolic biomarkers [14]. Adding derived indices to the raw variables changed the AUC by only 0.004, suggesting limited incremental information once their component counts were modeled directly.

Lower sodium was among the most frequently retained model signals. Hyponatremia has been associated with complicated appendicitis, possibly through inflammation-driven non-osmotic antidiuretic hormone release and fluid shifts [9]. Although much of the evidence is pediatric, the present adult data support further evaluation across age groups.

Total bilirubin was also selected consistently. Hyperbilirubinemia may reflect endotoxemia and cytokine-mediated impairment of bile transport. In 574 adults, bilirubin, CRP, age, and delayed presentation formed a model with an AUC of 0.877 [30]. Bilirubin should nevertheless be interpreted within the overall clinical profile, not as a stand-alone test.

Heart rate and respiratory rate had the highest selection frequencies; temperature, pain severity, and rigidity were also retained consistently. These immediately available measures may capture physiological stress and local peritoneal irritation that are not fully represented by laboratory markers.

Although the LASSO model showed only a modest and statistically non-significant improvement in discrimination over the AIR score, decision-curve analysis provided additional information regarding potential clinical utility [31,32]. The LASSO model yielded greater net benefit than AIR across a substantial range of threshold probabilities, with the clearest separation between approximately 0.15 and 0.25. Nevertheless, this advantage was not uniform across all thresholds and diminished at higher predicted-risk levels. AIR therefore remains an attractive bedside tool because of its simplicity and transparency [25]. The potential value of the LASSO approach lies primarily in automated electronic integration of routinely available clinical and laboratory data rather than manual bedside calculation. Its additional complexity would only be justified after independent external validation, confirmation of calibration, and prospective demonstration that the observed net-benefit advantage translates into improved clinical decision-making [31,32].

The observed AUC is consistent with a 2026 synthesis of complicated-appendicitis models, which reported lower performance during external than development validation [33], and with an internally validated adult clinical–laboratory–CT model [26]. This pattern reinforces the need to distinguish apparent performance from validation performance.

Nested cross-validation separated penalty tuning from performance estimation, addressing a common source of optimism in appendicitis prediction studies [33,34]. Bootstrap predictor-selection reproducibility analysis described how often predictors were retained across resamples. Selection frequency, however, is not a causal measure and does not resolve uncertainty created by correlated predictors.

Discrimination was preserved in the 2025 temporal cohort, but calibration was less favorable. The calibration intercept of −0.383 indicates systematic overprediction of absolute risk, while the slope of 0.895 suggests modestly excessive prediction extremity. This discrepancy illustrates that preservation of rank-order discrimination does not necessarily imply preservation of absolute risk estimates over time. Potential explanations include temporal changes in case mix, referral patterns, admission characteristics, disease prevalence, or other aspects of clinical practice; however, these mechanisms could not be directly established from the present retrospective data. Moreover, the temporal cohort contained only 21 complicated cases, resulting in substantial uncertainty around calibration assessment. These findings support external validation in larger contemporary cohorts and, if similar calibration drift is confirmed, model recalibration before clinical implementation. Prospective confirmation of the observed decision-curve net benefit at clinically justified thresholds is also required before routine clinical use.

The outcome sensitivity analyses demonstrate that predictive performance depends materially on how complicated appendicitis is defined. Histopathologically complicated disease represented a narrower category nested within the surgeon-documented intraoperative classification. Discrimination was particularly high for the surgeon-classified intraoperative outcome, whereas performance for the broader macroscopic composite was more moderate. The near-perfect discrimination observed for the surgeon-documented outcome should be interpreted cautiously and should not be viewed as evidence of generalizability, because the same physiological, inflammatory, and peritoneal findings used as predictors may be closely aligned with the clinical severity ultimately observed and documented during surgery. Exclusion of negative appendectomies reduced the primary-model AUC from 0.861 to 0.840, indicating that these readily separable cases contributed modestly to discrimination but did not fully explain model performance. Differences between intraoperative and histopathological classifications of appendicitis severity have also been reported previously [19,35]. Surgeons assess the entire operative field, while histopathology evaluates the specimen; neither is a complete substitute for the other.

In a 2026 cohort of 2279 appendectomies, concordance between operative and histopathological grading was 74%, and operative grading was more closely related to immediate resource use [20]. This supports interpreting the two classifications as complementary rather than competing reference standards.

Complicated histopathology was associated with longer surgery, more antibiotics, delayed gastrointestinal recovery, longer hospitalization, intra-abdominal abscess, readmission, and major morbidity. Similar gradients have been reported across anatomical and pathological severity categories [16,17,18]. Akın et al. likewise found longer hospitalization and higher postoperative complication rates in histopathologically complicated appendicitis compared with phlegmonous and uncomplicated categories [36]. The association with any 30-day complication persisted after limited adjustment (Supplementary Table S5); however, in the three-category analysis it was not clearly separable from intraoperative severity, and residual confounding remains likely.

The 4.6% negative appendectomy rate reflects the surgical target population. Negative appendectomies were retained in the non-complicated group because the model addresses patients already selected for appendectomy; this choice should be considered when comparing performance with cohorts restricted to histologically confirmed appendicitis. Routine histopathology also identifies unexpected neoplasia and other diagnoses [21,37].

### 4.1. Strengths and Limitations

Strengths include a well-characterized cohort, restriction of the prediction model to preoperative variables, penalized estimation, nested cross-validation, detailed assessment of discrimination and calibration, bootstrap assessment of predictor-selection reproducibility, comparison with established clinical scores, repeated nested cross-validation, and same-center temporal evaluation. Linking model performance with operative, histopathological, and 30-day postoperative outcomes also provided clinical context for the histopathological category being predicted.

Several limitations should be considered. First, the retrospective single-center design introduces potential selection and information bias. Physical examination findings such as rebound tenderness, guarding, and rigidity, as well as intraoperative severity assessments, depended partly on clinician documentation and judgment and may therefore be subject to interobserver and documentation variability. In addition, the exact timing of individual preoperative vital-sign, examination, and laboratory measurements relative to analgesia, intravenous fluids, or antibiotic administration could not be reconstructed consistently from the retrospective records. Consequently, some predictor values may have been influenced by clinical management before surgery.

Second, the effective sample size for model development was limited, with 111 primary-outcome events for 50 candidate predictors. Although LASSO penalization and nested cross-validation reduce overfitting and optimism, they do not fully compensate for limited information relative to model complexity. Predictor-selection frequencies and individual coefficient estimates should therefore be interpreted cautiously, particularly for infrequent binary predictors and correlated variables. In addition, the bootstrap confidence interval around the primary out-of-fold AUC was conditional on the predictions generated by the original nested cross-validation fits and did not incorporate the full uncertainty associated with model retraining. Repeated nested cross-validation demonstrated expected variation across alternative fold allocations and was therefore used as a complementary assessment of performance variability rather than as a substitute for independent external validation. In addition, continuous predictors were entered as linear terms on the log-odds scale, and linearity was not formally assessed, for example using restricted cubic splines or fractional polynomials. Clinically plausible nonlinear relationships, such as risk increasing only above a certain level or reaching a plateau, may therefore have been misspecified; this could have contributed to the non-uniform calibration observed across the predicted-risk range and may have affected the apparent contribution of individual predictors. Bootstrap selection frequencies were also estimated at a fixed penalty and therefore do not reflect the full variability of the model-building process. Future development studies should evaluate flexible functional forms, at least for the most influential continuous predictors.

Third, temporal evaluation was performed at the same institution and included only 21 patients with histopathologically complicated appendicitis. Consequently, the precision of discrimination and especially calibration estimates in the temporal cohort was limited. The negative calibration intercept observed in the 2025 cohort indicated systematic overprediction, emphasizing that absolute risk estimates may require recalibration before application in other periods or settings. This same-center temporal assessment therefore does not establish geographic or broader external transportability.

Fourth, imaging was performed according to routine clinical indications rather than a standardized study protocol and was deliberately excluded from the primary clinical–laboratory prediction model. Consequently, the present analysis cannot determine the incremental predictive value of CT or ultrasonography relative to the clinical–laboratory model, and direct comparison with imaging-inclusive prediction strategies remains necessary.

Finally, the study population consisted exclusively of adults who had already been selected for appendectomy. Accordingly, the model estimates the probability of histopathologically complicated appendicitis conditional on surgical selection and should not be extrapolated to the broader population of patients presenting with suspected appendicitis or used to determine eligibility for nonoperative treatment. A development-derived probability threshold of 0.176 achieved 95.6% sensitivity in the 2021–2024 development cohort but only 90.5% sensitivity when applied unchanged to the 2025 cohort, underscoring that this exploratory threshold is not a validated clinical rule-out criterion.

Fairness-specific modeling and formal subgroup performance analyses were not performed. The single-center sample and limited number of outcome events restricted reliable assessment of differential model performance across sociodemographic groups; potential health-equity implications should therefore be examined in larger and more diverse external cohorts.

### 4.2. Clinical Implications and Future Directions

The model integrates routinely available physiological, examination, hematological, and biochemical signals, supporting multidimensional risk assessment rather than reliance on isolated cutoffs. Its potential clinical role should be defined through independent multicenter validation and prospective evaluation of decision impact. The model has not been externally validated or implemented as a clinical tool; consequently, no clinical user interface or validated strategy for handling unavailable predictor values has been specified, and workflow/user-interaction requirements should be defined only after external validation and prospective implementation testing.

Next steps are independent multicenter validation, assessment and potential recalibration across settings, and prospective confirmation of the observed net-benefit findings at clinically justified thresholds. In settings where CT is routinely incorporated into appendicitis assessment, the present model should be considered an imaging-independent adjunct rather than a substitute for radiological evaluation. Future studies should directly compare and combine clinical–laboratory and imaging-inclusive models to quantify the incremental predictive value of radiological information.

## 5. Conclusions

Histopathologically complicated acute appendicitis was associated with a distinct clinical, physiological, and inflammatory profile and with less favorable short-term surgical outcomes. The LASSO model based on routinely available preoperative clinical and laboratory variables achieved good internally validated discrimination; however, its improvement over the AIR score was small and not statistically significant. Same-center temporal evaluation showed similar discrimination but also evidence of overprediction, with limited precision because only 21 outcome events occurred in the 2025 cohort. Accordingly, the model should not be considered a replacement for established clinical scores or be used for clinical decision-making without further validation. Independent multicenter validation, assessment of calibration and potential recalibration, and comparison with imaging-inclusive strategies are required before clinical implementation.

## Figures and Tables

**Table 1:** Baseline demographic and clinical characteristics according to histopathological severity.

Variable	Non-Complicated (*n* = 389)	Complicated (*n* = 111)	Effect Estimate (95% CI)	*p* Value
Age, years	34 (27–44)	35 (25–45)	+1.0 years (−3.0 to 6.0)	0.959
Male sex, *n* (%)	210 (54.0)	66 (59.5)	OR 1.25 (0.81–1.92)	0.306
BMI, kg/m^2^	25.9 (23.5–28.4)	25.5 (23.2–28.6)	−0.4 kg/m^2^ (−1.1 to 0.6)	0.430
Charlson Comorbidity Index	0 (0–0)	0 (0–1)	0.0 (0.0–0.0)	0.203
ASA physical status class	1 (1–1)	1 (1–2)	0.0 (0.0–0.0)	0.403
Diabetes mellitus, *n* (%)	29 (7.5)	17 (15.3)	OR 2.25 (1.18–4.26)	0.011
Hypertension, *n* (%)	58 (14.9)	16 (14.4)	OR 0.96 (0.53–1.75)	0.897
Smoking, *n* (%)	97 (24.9)	31 (27.9)	OR 1.17 (0.73–1.87)	0.524
Previous abdominal surgery, *n* (%)	58 (14.9)	16 (14.4)	OR 0.96 (0.53–1.75)	0.897
Referral from another center, *n* (%)	68 (17.5)	26 (23.4)	OR 1.44 (0.87–2.41)	0.158
Prehospital antibiotic use, *n* (%)	42 (10.8)	10 (9.0)	OR 0.82 (0.40–1.69)	0.586
Symptom duration, h	20.3 (13.7–30.8)	36.0 (25.6–56.0)	+15.7 h (11.5–20.4)	<0.001
Pain VAS score	7 (6–8)	8 (7–8)	+1.0 (0.0–1.0)	<0.001
Temperature, °C	37.2 (37.0–37.6)	37.7 (37.4–38.0)	+0.5 °C (0.4–0.6)	<0.001
Heart rate, beats/min	85 (78–91)	93 (86.5–100)	+8 beats/min (6–12)	<0.001
Respiratory rate, breaths/min	18 (16–20)	21 (19–22)	+3 breaths/min (2–3)	<0.001
Systolic blood pressure, mmHg	124 (112–135)	122 (111.5–134)	−2 mmHg (−8 to 2)	0.174
Shock index	0.69 (0.61–0.78)	0.78 (0.70–0.87)	+0.09 (0.06–0.14)	<0.001
Pain migration, *n* (%)	197 (50.6)	56 (50.5)	OR 0.99 (0.65–1.51)	0.971
Anorexia, *n* (%)	240 (61.7)	72 (64.9)	OR 1.15 (0.74–1.78)	0.543
Nausea/vomiting, *n* (%)	204 (52.4)	68 (61.3)	OR 1.43 (0.93–2.21)	0.100
Rebound tenderness, *n* (%)	244 (62.7)	93 (83.8)	OR 3.07 (1.78–5.29)	<0.001
Guarding, *n* (%)	141 (36.2)	71 (64.0)	OR 3.12 (2.01–4.84)	<0.001
Rigidity, *n* (%)	19 (4.9)	24 (21.6)	OR 5.37 (2.82–10.25)	<0.001

Note. Data are presented as median (interquartile range) or *n* (%). For continuous variables, effect estimates represent differences in medians (complicated minus non-complicated) with bootstrap 95% confidence intervals based on 10,000 resamples. For binary variables, effect estimates are odds ratios with 95% confidence intervals, with the non-complicated group as the reference. BMI, body mass index; ASA, American Society of Anesthesiologists; VAS, visual analogue scale; OR, odds ratio; CI, confidence interval.

**Table 2:** Surgical and 30-day postoperative outcomes according to histopathological severity.

Outcome	Non-Complicated (*n* = 389)	Complicated (*n* = 111)	Effect Estimate (95% CI)	*p* Value	q Value
Operation duration, min	52.0 (40.0–62.0)	71.0 (58.5–79.5)	+19.0 min (16.0–22.0)	<0.001	<0.001
Postoperative antibiotic duration, days	1 (0–2)	4 (3–4)	+3.0 days (2.0–3.0)	<0.001	<0.001
Time to oral intake, h	10.9 (6.9–15.7)	18.6 (16.0–22.8)	+7.7 h (6.5–9.6)	<0.001	<0.001
Time to first flatus, h	22.4 (16.9–28.4)	32.3 (27.5–37.2)	+9.9 h (7.7–11.9)	<0.001	<0.001
Length of hospital stay, days	2.0 (1.3–3.1)	3.5 (2.5–5.3)	+1.5 days (1.1–2.1)	<0.001	<0.001
Any 30-day complication, *n* (%)	31 (8.0)	17 (15.3)	OR 2.09 (1.11–3.94)	0.020	0.044
Superficial SSI, *n* (%)	11 (2.8)	6 (5.4)	OR 1.96 (0.71–5.43)	0.231	0.302
Deep SSI, *n* (%)	2 (0.5)	1 (0.9)	OR 1.76 (0.16–19.58)	0.530	0.563
Intra-abdominal abscess, *n* (%)	7 (1.8)	8 (7.2)	OR 4.24 (1.50–11.96)	0.007	0.018
Postoperative ileus, *n* (%)	5 (1.3)	3 (2.7)	OR 2.13 (0.50–9.07)	0.384	0.436
Percutaneous drainage, *n* (%)	3 (0.8)	4 (3.6)	OR 4.81 (1.06–21.82)	0.046	0.072
ICU admission, *n* (%)	1 (0.3)	2 (1.8)	OR 7.12 (0.64–79.26)	0.125	0.178
Emergency department revisit, *n* (%)	10 (2.6)	11 (9.9)	OR 4.17 (1.72–10.09)	0.002	0.005
30-day readmission, *n* (%)	6 (1.5)	6 (5.4)	OR 3.65 (1.15–11.54)	0.030	0.051
Reoperation, *n* (%)	3 (0.8)	2 (1.8)	OR 2.36 (0.39–14.31)	0.308	0.374
Major morbidity, *n* (%)	8 (2.1)	7 (6.3)	OR 3.21 (1.14–9.04)	0.029	0.051
30-day mortality, *n* (%)	1 (0.3)	0 (0.0)	—	1.000	1.000

Note. Continuous outcomes are reported as median (interquartile range), and effect estimates represent differences in medians (complicated minus non-complicated) with bootstrap 95% confidence intervals based on 10,000 resamples. Binary outcomes are reported as *n* (%), with odds ratios (ORs) and 95% confidence intervals. q values were calculated using the Benjamini–Hochberg false-discovery-rate procedure across the 17 secondary surgical and postoperative outcome comparisons. Individual postoperative complication categories were not mutually exclusive; therefore, a single patient could contribute to more than one component complication. SSI, surgical-site infection; ICU, intensive care unit.

**Table 3:** Internally validated predictive performance for histopathologically complicated appendicitis.

Performance Measure	Result
LASSO nested-CV AUC	0.861 (conditional bootstrap 95% CI, 0.828–0.892)
Brier score	0.129
Null Brier score	0.173
Scaled Brier score	0.253
Calibration intercept	−0.158 (95% CI, −0.445 to 0.129)
Calibration slope	0.898 (95% CI, 0.719–1.078)
AIR score AUC	0.841
LASSO vs. AIR, ΔAUC	0.020 (95% CI, −0.011 to 0.054; *p* = 0.221)
Alvarado score AUC	0.743
LASSO vs. Alvarado, ΔAUC	0.117 (95% CI, 0.067–0.169; *p* < 0.001)

AUC, area under the receiver operating characteristic curve; AIR, Appendicitis Inflammatory Response; CI, confidence interval; CV, cross-validation; LASSO, least absolute shrinkage and selection operator. The null Brier score was calculated as π(1 − π), where π was the observed prevalence of histopathologically complicated appendicitis (111/500, 22.2%). The scaled Brier score was calculated as 1 − (model Brier score/null Brier score), with higher values indicating greater improvement over the null prediction model. AUC differences were estimated using paired patient-level resampling of predictions and scores obtained from the same patients. The confidence interval for the primary cross-validated AUC was obtained by patient-level bootstrap resampling of fixed pooled out-of-fold predictions and should therefore be interpreted as conditional on the fitted nested-cross-validation models; it does not include uncertainty arising from model retraining or alternative fold allocations. The latter was examined separately by repeated nested cross-validation. For the AIR and Alvarado scores, uncertainty is expressed through the paired AUC differences relative to the LASSO model, which account for the correlation between predictions obtained from the same patients; AUCs with 95% CIs for all three approaches in the 2025 cohort are reported in Supplementary Table S4.

**Table 4:** Frequently retained LASSO predictors: bootstrap selection frequency, standardized coefficient, and direction of association.

Predictor	Selection Frequency (%)	Standardized Coefficient	Direction of Association
Heart rate	99.5	0.415	Higher risk
Respiratory rate	99.4	0.368	Higher risk
Serum sodium	97.5	−0.270	Lower risk
Absolute lymphocyte count	96.6	−0.299	Lower risk
Neutrophils (%)	96.0	0.258	Higher risk
Abdominal rigidity	95.1	0.198	Higher risk
Total bilirubin	93.3	0.205	Higher risk
Age	93.2	−0.255	Lower risk
Serum glucose	93.2	0.193	Higher risk
Pain VAS score	92.7	0.189	Higher risk
Diarrhea	92.4	0.147	Higher risk
Systolic blood pressure	89.9	−0.171	Lower risk
BMI	87.7	−0.177	Lower risk
Body temperature	85.2	0.173	Higher risk
Rebound tenderness	80.3	0.112	Higher risk

Note. Selection frequency represents the percentage of 1000 bootstrap samples in which the predictor retained a non-zero LASSO coefficient using the penalty selected in the complete analytical cohort. Because the penalty parameter was held fixed across bootstrap samples, these frequencies are descriptive and may overestimate selection reproducibility. They should not be interpreted as formal measures of predictor importance. Complete frequencies for all 50 candidate predictors are provided in Supplementary Table S3. VAS, visual analogue scale; BMI, body mass index. Standardized coefficients and directions of association are taken from the single final LASSO model fitted to the complete analytical cohort (Supplementary Table S6) and represent conditional associations within the multivariable penalized model; they may therefore differ from univariable comparisons (e.g., age). “Higher risk” indicates that a higher value, or presence of the finding, was associated with a higher predicted probability of histopathologically complicated appendicitis; “Lower risk” indicates the opposite. No selection-frequency threshold was used to classify predictors as stable; the 15 predictors with the highest selection frequencies are shown.

## Data Availability

The data are not publicly available because they contain potentially identifiable clinical information and are subject to institutional and ethical restrictions. De-identified data and analysis code may be made available by the corresponding author on reasonable request, subject to approval by the responsible institution and ethics committee.

## Supplementary Materials

The following supporting information can be downloaded at https://www.mdpi.com/article/10.3390/jcm15208063/s1. Table S1: Detailed laboratory parameters and inflammatory indices according to histopathological severity; Table S2: Sensitivity analysis evaluating the incremental value of derived inflammatory indices; Table S3: Complete descriptive bootstrap selection frequencies for the 50 candidate predictors; Table S4: Same-center temporal evaluation of the LASSO prediction model; Table S5: Limited-adjustment sensitivity model for any 30-day postoperative complication; Table S6: Final LASSO model specification for prediction of histopathologically complicated appendicitis; Table S7: Sensitivity analyses using alternative definitions of complicated appendicitis and excluding negative appendectomies; Table S8: Three-category analysis of intraoperative and histopathological severity and postoperative outcomes; Table S9: Sensitivity of internally validated model performance to the inner-loop tuning criterion; Table S10: Repeated nested cross-validation sensitivity analysis; Figure S1: Receiver operating characteristic curve for same-center temporal evaluation in the 2025 cohort. A completed TRIPOD+AI reporting checklist is provided as a separate supplementary file.
