# Supplementary material for: Clinical and Laboratory Prediction of Histopathologically Complicated Acute Appendicitis and Associated Surgical Outcomes: A Retrospective Cohort Study
Source: J Clin Med. 2026 Oct 17;15(20):8063. doi: 10.3390/jcm15208063 (PMC13706473; doi:10.3390/jcm15208063)
Supplement: Supplementary file 1 [file jcm-15-08063-s001.zip › JCM_Supplementary_Materials_R1.pdf]

Journal of
Clinical Medicine

MDPI

Supplementary Materials

Clinical and Laboratory Prediction of Histopathologically Complicated Acute Appendicitis and Associated Surgical Outcomes: A Retrospective Cohort Study

Volkan Gökmen, Eva Kajti, Dursun Alper Yılmaz and Adem Aslan

These supplementary materials provide detailed laboratory comparisons, sensitivity analyses, complete bootstrap predictor-selection results, same-center temporal evaluation, the final LASSO model specification, alternative-outcome analyses, postoperative-outcome analyses, and nested cross-validation sensitivity analyses referenced in the main manuscript.

Supplementary Table S1. Detailed laboratory parameters and inflammatory indices according to histopathological severity.

Variable	Non-complicated (n=389)	Complicated (n=111)	p value
WBC, ×10³/µL	13.34 (11.23–15.17)	16.21 (13.74–17.78)	<0.001
Neutrophils, %	78.4 (74.5–82.8)	84.9 (81.6–88.7)	<0.001
Absolute neutrophil count, ×10³/µL	10.25 (8.72–12.26)	13.49 (11.46–15.35)	<0.001
Absolute lymphocyte count, ×10³/µL	1.52 (1.17–1.87)	1.04 (0.71–1.50)	<0.001
Platelet count, ×10³/µL	263 (227–297)	282 (246–308)	0.005
CRP, mg/L	29.1 (14.5–56.1)	79.2 (46.7–129.1)	<0.001
Glucose, mg/dL	108 (94–120)	118 (108–134)	<0.001
Creatinine, mg/dL	0.88 (0.78–1.00)	0.92 (0.81–1.03)	0.053
Sodium, mmol/L	138.0 (136.5–139.6)	136.1 (134.6–137.9)	<0.001
Total bilirubin, mg/dL	0.72 (0.55–0.98)	1.05 (0.84–1.38)	<0.001
Albumin, g/dL*	4.14 (3.88–4.39)	3.92 (3.73–4.16)	<0.001
NLR	6.76 (5.01–9.41)	12.98 (8.65–19.74)	<0.001
PLR	171.5 (134.9–237.0)	263.0 (188.7–407.2)	<0.001
MLR	0.42 (0.32–0.60)	0.68 (0.50–1.03)	<0.001
SII	1757 (1261–2644)	3613 (2324–5538)	<0.001
SIRI	4.32 (2.91–6.52)	9.12 (6.03–13.78)	<0.001
CRP/albumin ratio*	6.88 (3.67–13.99)	19.48 (11.45–32.65)	<0.001
Alvarado score	7 (6–8)	9 (8–9)	<0.001

Variable	Non-complicated (n=389)	Complicated (n=111)	p value
AIR score	6 (5–7)	8 (8–9)	<0.001

Note. Albumin-derived variables were available in 439 patients. AIR, Appendicitis Inflammatory Response; CRP, C-reactive protein; MLR, monocyte-to-lymphocyte ratio; NLR, neutrophil-to-lymphocyte ratio; PLR, platelet-to-lymphocyte ratio; SII, systemic immune-inflammation index; SIRI, systemic inflammation response index; WBC, white blood cell count.

Supplementary Table S2. Sensitivity analysis evaluating the incremental value of derived inflammatory indices.

Model	Predictor set	Cross-validated AUC	Brier score	Comparison with primary model
Primary LASSO	50 raw clinical and laboratory candidate predictors	0.861	0.129	Reference
Extended LASSO	Primary predictor set + NLR, PLR, MLR, SII, and SIRI	0.865	0.128	ΔAUC 0.004 (95% CI −0.003 to 0.013); p=0.302

Note. Both models were evaluated using the same nested stratified cross-validation framework. The addition of derived inflammatory indices did not materially improve discrimination. AUC, area under the receiver operating characteristic curve; LASSO, least absolute shrinkage and selection operator.

Supplementary Table S3. Complete descriptive bootstrap selection frequencies for the 50 candidate predictors.

Predictor	Selection frequency (%)	Standardized LASSO coefficient
Heart rate	99.5	0.4151
Respiratory rate	99.4	0.3684
Serum sodium	97.5	-0.2695
Absolute lymphocyte count	96.6	-0.2993
Neutrophils (%)	96.0	0.2576
Abdominal rigidity	95.1	0.1979
Total bilirubin	93.3	0.2050
Age	93.2	-0.2547
Serum glucose	93.2	0.1932
Pain VAS score	92.7	0.1887
Diarrhea	92.4	0.1468
Systolic blood pressure	89.9	-0.1709
BMI	87.7	-0.1768
Body temperature	85.2	0.1729

Predictor	Selection frequency (%)	Standardized LASSO coefficient
Rebound tenderness	80.3	0.1118
Anticoagulant use	79.2	-0.1255
Mean platelet volume	69.5	0.0730
Absolute monocyte count	69.1	0.0593
Coronary artery disease	66.2	0.0601
Creatinine	66.1	0.0625
Altered mental status	65.4	0.0000
Nausea/vomiting	63.6	0.0467
Pain migration	60.7	0.0505
Antiplatelet use	57.3	0.0439
Constipation	56.4	0.0321
Diabetes mellitus	56.1	0.0527
Absolute neutrophil count	54.9	0.2025
Peripheral oxygen saturation	54.7	-0.0177
Symptom duration	52.2	0.0000
Hypertension	50.7	0.0000
Guarding	50.2	0.0146
Referral from another center	48.5	0.0000
Right lower-quadrant tenderness	46.8	0.0000
Male sex	44.9	0.0000
Chronic kidney disease	42.9	-0.0023
Platelet count	42.4	0.0000
Previous abdominal surgery	41.7	0.0000
Smoking	41.5	0.0000
White blood cell count	41.5	0.0000
Immunosuppression	39.2	-0.0065
Anorexia	38.7	0.0000
Diastolic blood pressure	35.6	0.0000
Red cell distribution width	35.5	0.0000
Dysuria	35.3	0.0000
Hemoglobin	35.1	0.0000

Predictor	Selection frequency (%)	Standardized LASSO coefficient
COPD	34.9	0.0000
Prehospital antibiotic exposure	34.7	0.0000
C-reactive protein	34.0	0.0000
Charlson Comorbidity Index	11.7	0.0000
ASA class	11.5	0.0000

Note. Selection frequency is the percentage of 1,000 bootstrap samples in which the predictor retained a non-zero LASSO coefficient using the penalty selected in the complete analytical cohort. The penalty was held fixed across bootstrap samples; therefore, these frequencies are descriptive and may overestimate selection reproducibility. No selection-frequency threshold was used to define predictors as "stable." Coefficients are standardized and are provided for descriptive model interpretation rather than causal inference. The standardized coefficient column reports the coefficients of the single final LASSO model fitted to the complete analytical cohort (C = 0.17782794; Supplementary Table S6), whereas selection frequencies were obtained from 1,000 bootstrap refits. The two columns therefore originate from different fits. A predictor whose contribution lies close to the penalization boundary, or overlaps with that of correlated predictors, may be retained in a substantial proportion of bootstrap resamples yet be shrunk exactly to zero in the complete-cohort fit (e.g., altered mental status, symptom duration, and C-reactive protein); conversely, a predictor may have a non-zero coefficient despite a moderate selection frequency.

Supplementary Table S4. Same-center temporal evaluation of the LASSO prediction model.

Measure	Same-center temporal evaluation result
**Development period**	2021–2024
**Development cohort, n**	399
**Complicated cases, development**	90 (22.6%)
**Evaluation period**	2025
**Evaluation cohort, n**	101
**Complicated cases, evaluation**	21 (20.8%)
**LASSO AUC**	0.859 (95% CI, 0.783–0.926)
**AIR AUC**	0.872 (95% CI, 0.786–0.942)
**Alvarado AUC**	0.760 (95% CI, 0.637–0.871)
**Brier score**	0.131
**Null Brier score**	0.165
**Scaled Brier score**	0.205
**Calibration intercept**	−0.383

**Calibration slope**	0.895
**Development-derived high-sensitivity threshold**	0.176
**Development sensitivity at threshold**	95.6%
**Development specificity at threshold**	73.1%
**2025 sensitivity at fixed threshold**	90.5% (19/21)
**2025 specificity at fixed threshold**	73.8% (59/80)
**2025 PPV**	47.5%
**2025 NPV**	96.7%

Note. The 2021–2024 development cohort and the 2025 evaluation cohort were drawn from the same institution and used the same eligibility criteria, outcome definition, candidate-predictor definitions, and data-collection framework; they differed primarily by calendar period. The high-sensitivity threshold was selected using only out-of-fold predictions from the 2021–2024 development cohort and was applied unchanged to the 2025 cohort. No 2025 observations contributed to scaling, penalty selection, model fitting, or threshold selection. AUC confidence intervals were estimated using patient-level bootstrap resampling. AIR, Appendicitis Inflammatory Response; AUC, area under the receiver operating characteristic curve; PPV, positive predictive value; NPV, negative predictive value.

Supplementary Table S5. Limited-adjustment sensitivity model for any 30-day postoperative complication.

Predictor	Adjusted OR	95% CI	p value
Histopathologically complicated appendicitis	2.20	1.14–4.25	0.018
Age, per year	1.03	1.00–1.05	0.021
Male sex	1.79	0.94–3.44	0.078
Diabetes mellitus	0.31	0.08–1.20	0.090
ASA class, per category	1.25	0.68–2.28	0.473

Note. This secondary model was retained as a limited-adjustment sensitivity analysis and included histopathologically complicated appendicitis, age, sex, diabetes mellitus, and ASA class. Because only 48 patients experienced a 30-day postoperative complication, the model was intentionally restricted and should be interpreted as exploratory. The expanded three-category severity analysis, which more directly accounts for intraoperative classification, is reported in Supplementary Table S8. OR, odds ratio; ASA, American Society of Anesthesiologists.

Supplementary Table S6. Final LASSO model specification for prediction of histopathologically complicated appendicitis.

Predictor	Mean	SD	Standardized β	Raw-scale β
Age, years	37.5000	15.0501	−0.254763	−0.016928
BMI, kg/m²	26.0044	3.8511	−0.176809	−0.045911

**Diabetes mellitus**	0.0920	0.2890	0.052766	0.182565
**Coronary artery disease**	0.0280	0.1650	0.060115	0.364391
**Chronic kidney disease**	0.0220	0.1467	−0.002300	−0.015682
**Immunosuppression**	0.0260	0.1591	−0.006486	−0.040757
**Anticoagulant use**	0.0140	0.1175	−0.125483	−1.068031
**Antiplatelet use**	0.0280	0.1650	0.043936	0.266323
**Pain VAS score**	6.9620	1.3478	0.188736	0.140033
**Temperature, °C**	37.3920	0.4977	0.172905	0.347386
**Heart rate, beats/min**	86.9320	10.8285	0.415090	0.038333
**Respiratory rate, breaths/min**	18.6140	2.9019	0.368441	0.126966
**Systolic blood pressure, mmHg**	122.8400	15.6626	−0.170917	−0.010912
**SpO_2, %**	97.2560	1.1342	−0.017709	−0.015613
**Pain migration**	0.5060	0.5000	0.050453	0.100912
**Nausea/vomiting**	0.5440	0.4981	0.046756	0.093877
**Rebound tenderness**	0.6740	0.4687	0.111757	0.238417
**Guarding**	0.4240	0.4942	0.014562	0.029465
**Rigidity**	0.0860	0.2804	0.197940	0.706009
**Diarrhea**	0.0860	0.2804	0.146831	0.523715
**Constipation**	0.1120	0.3154	0.032109	0.101816
**Neutrophils, %**	80.2702	6.2259	0.257568	0.041370
**Absolute neutrophil count, $\times10^3/\mu L$**	11.2398	3.0472	0.202527	0.066463
**Absolute lymphocyte count, $\times10^3/\mu L$**	1.4290	0.5571	−0.299306	−0.537297
**Absolute monocyte count, $\times10^3/\mu L$**	0.6749	0.1850	0.059342	0.320698
**Mean platelet volume, fL**	9.5116	0.9108	0.073031	0.080183
**Glucose, mg/dL**	110.6620	20.1437	0.193162	0.009589
**Creatinine, mg/dL**	0.8980	0.1721	0.062467	0.362984
**Sodium, mmol/L**	137.5824	2.5526	−0.269542	−0.105596
**Total bilirubin, mg/dL**	0.8904	0.4196	0.205009	0.488580

Model specification. The final full-cohort LASSO model retained 30 predictors with non-zero coefficients and used an inverse regularization parameter of C = 0.17782794. For the standardized model, the linear predictor is

$\eta = -1.781374 + \Sigma\beta jzj$,

where $zj = (xj - \mu j)/\sigma j$, and μj, σj, and βj are the predictor-specific mean, standard deviation, and standardized LASSO coefficient reported above. The predicted probability of histopathologically complicated appendicitis is

$p = 1/[1 + \exp(-\eta)]$.

Binary predictors were coded as 0 or 1 and were subjected to the same centering and scaling procedure as continuous predictors. Predictors with coefficients shrunk exactly to zero are omitted from the table. Equivalently, using the raw-scale coefficients reported in the final column, $\eta = -8.737510 + \Sigma\gamma jxj$, where γj denotes the corresponding raw-scale coefficient; the predicted probability is again $p = 1/[1 + \exp(-\eta)]$. This complete-cohort fit is provided for reproducibility and its apparent performance was not used as an estimate of validated model performance.

Supplementary Table S7. Sensitivity analyses using alternative definitions of complicated appendicitis and excluding negative appendectomies.

Sensitivity outcome	N	Events, n (%)	LASSO AUC (95% CI)	Brier score	AIR AUC	Alvarado AUC
**Primary histopathological definition**	500	111 (22.2)	0.861 (0.828–0.892)	0.129	0.841	0.743
**Surgeon-documented intraoperative definition**	500	192 (38.4)	0.998 (0.997–1.000)	0.016	0.947	0.797
**Macroscopic composite definition**	500	222 (44.4)	0.885 (0.851–0.917)	0.114	0.858	0.735
**Primary definition excluding negative appendectomies**	477	111 (23.3)	0.840 (0.802–0.880)	0.135	0.833	0.734

Supplementary Table S8. Three-category analysis of intraoperative and histopathological severity and postoperative outcomes.

Outcome	Uncomplicated by both (n=308)	Intraoperative-only complicated (n=81)	Complicated by both (n=111)	Global p value
**Operation duration, min**	48.0 (38.0–58.0)	68.0 (58.0–79.0)	71.0 (58.5–79.5)	<0.001
**Postoperative antibiotic duration, days**	1 (0–1)	4 (3–5)	4 (3–4)	<0.001
**Time to oral intake, h**	9.5 (6.1–12.9)	18.0 (14.4–21.9)	18.6 (16.0–22.8)	<0.001
**Time to first flatus, h**	20.2 (15.6–24.7)	33.8 (29.0–38.6)	32.3 (27.5–37.2)	<0.001
**Length of hospital stay, days**	1.8 (1.2–2.5)	3.7 (3.1–4.8)	3.5 (2.5–5.3)	<0.001
**Any 30-day complication, n (%)**	20 (6.5)	11 (13.6)	17 (15.3)	**0.011**
**Intra-abdominal abscess, n (%)**	3 (1.0)	4 (4.9)	8 (7.2)	**0.001**
**Emergency department revisit, n (%)**	6 (1.9)	4 (4.9)	11 (9.9)	**0.002**
**30-day readmission, n (%)**	3 (1.0)	3 (3.7)	6 (5.4)	**0.015**

https://doi.org/10.3390/jcm15208063

**Major morbidity (Clavien–Dindo ≥III), n (%)**	5 (1.6)	3 (3.7)	7 (6.3)	**0.027**

Panel B. Exploratory multivariable logistic regression for any 30-day postoperative complication

Predictor	Adjusted OR	95% CI	p value
**Uncomplicated by both**	Reference	—	—
**Intraoperative-only complicated**	**1.83**	0.80–4.21	0.155
**Complicated by both**	**2.53**	1.19–5.37	**0.016**
**Age, per year**	1.03	1.00–1.05	0.026
**Male sex**	1.77	0.92–3.43	0.089
**Diabetes mellitus**	0.32	0.08–1.23	0.096
**ASA class, per category**	1.22	0.67–2.25	0.516
**Initial laparoscopic approach**	0.80	0.32–2.00	0.637
**Conversion to open surgery**	3.72	0.35–40.09	0.278
**Resident + attending surgeon**	0.86	0.45–1.63	0.643
**Time from admission to surgery, per hour**	0.99	0.92–1.07	0.825
**Referral from another center**	0.79	0.35–1.82	0.583
**Both complicated vs. intraoperative-only complicated***	**1.38**	0.59–3.24	0.459

Note. The three mutually exclusive severity categories were defined as uncomplicated by both intraoperative and histopathological assessment, complicated intraoperatively but non-complicated histopathologically, and complicated by both assessments. Continuous variables are presented as median (interquartile range). Global comparisons for continuous outcomes used the Kruskal–Wallis test; exact tests were used for sparse categorical outcomes where appropriate. The multivariable model was adjusted for age, sex, diabetes mellitus, ASA class, initial operative approach, conversion to open surgery, surgeon level, time from admission to surgery, and referral status. Because only 48 patients experienced the outcome, the adjusted analysis was considered exploratory. *The final row represents a post-estimation contrast between the both-complicated and intraoperative-only groups. OR, odds ratio; CI, confidence interval; ASA, American Society of Anesthesiologists.

Supplementary Table S9. Sensitivity of internally validated model performance to the inner-loop tuning criterion.

Inner-loop criterion	OOF AUC	Brier score	Log-loss	Calibration intercept	Calibration slope
**AUC (primary analysis)**	0.861	0.129	0.386	−0.158	0.898

**Logistic log-loss (sensitivity analysis)**	0.863	0.128	0.381	−0.123	0.950

Note. The complete nested cross-validation procedure was repeated with the inner-loop optimization criterion changed from AUC to logistic log-loss. Candidate predictors, preprocessing, outer and inner cross-validation structure, and all other model specifications were unchanged. OOF, out-of-fold; AUC, area under the receiver operating characteristic curve.

Supplementary Table S10. Repeated nested cross-validation sensitivity analysis.

Repetition	OOF AUC	Brier score	Calibration intercept	Calibration slope
**1**	0.861	0.129	−0.158	0.898
**2**	0.864	0.127	−0.095	1.136
**3**	0.854	0.133	−0.215	1.008
**4**	0.862	0.127	−0.146	1.003
**5**	0.854	0.130	−0.160	1.006
**6**	0.844	0.131	−0.148	1.013
**7**	0.847	0.132	−0.106	1.077
**8**	0.852	0.132	−0.235	0.920
**9**	0.862	0.127	−0.026	1.209
**10**	0.863	0.129	−0.161	1.028
**Mean (SD)**	**0.856 (0.007)**	**0.130 (0.002)**	**−0.145 (0.059)**	**1.030 (0.092)**

Note. The complete nested cross-validation framework was repeated 10 times using different random partitions. Each repetition used stratified 10-fold outer cross-validation and stratified 5-fold inner cross-validation, with preprocessing performed within training folds and AUC used for inner-loop penalty selection. OOF, out-of-fold; AUC, area under the receiver operating characteristic curve; SD, standard deviation.

https://doi.org/10.3390/jcm15208063

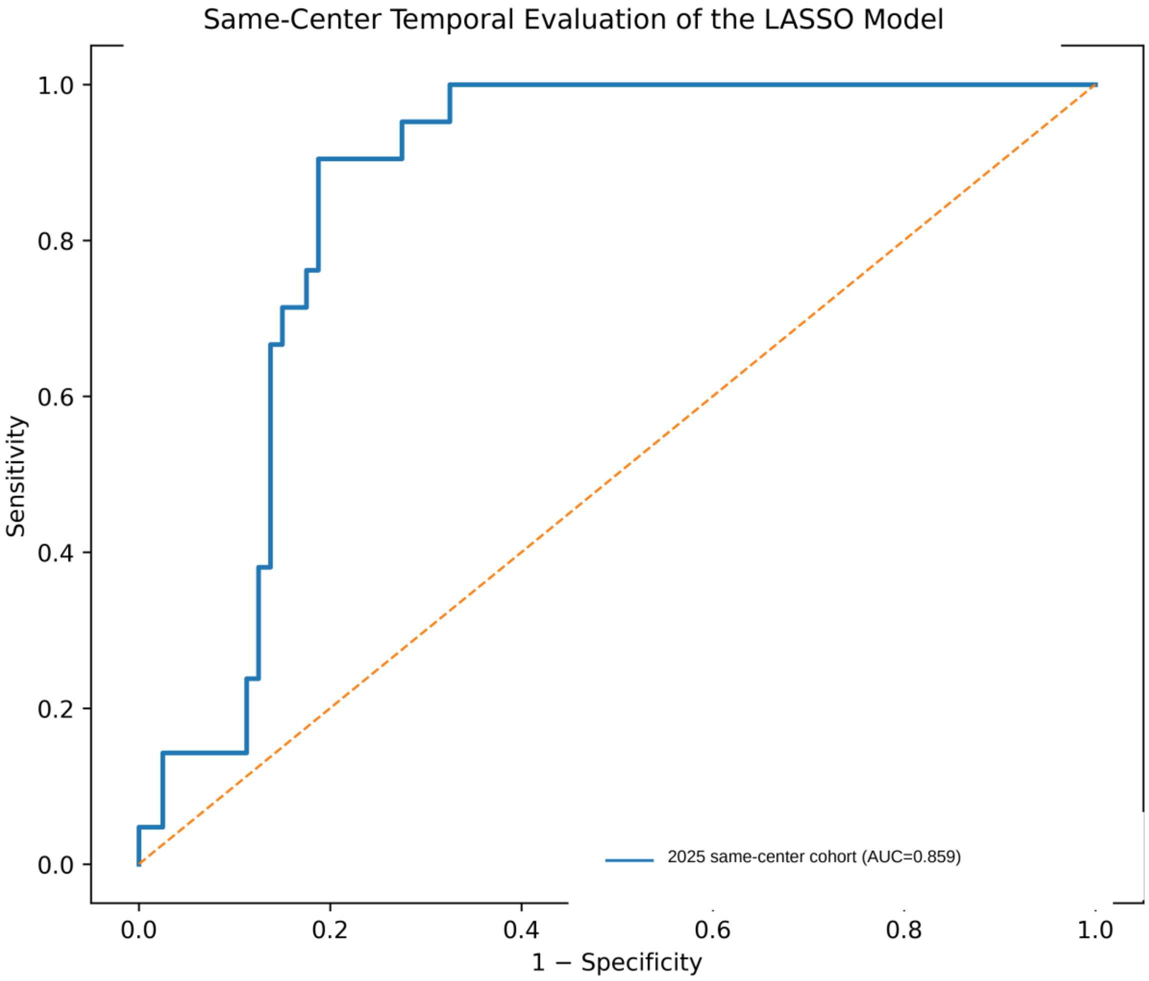

Supplementary Figure S1. Receiver operating characteristic curve for same-center temporal evaluation in the 2025 cohort.

The model developed using 2021–2024 data achieved an AUC of 0.859 (95% CI, 0.783–0.926) in the later same-center 2025 cohort.
