# Supplementary material for: Clinical and Laboratory Prediction of Histopathologically Complicated Acute Appendicitis and Associated Surgical Outcomes: A Retrospective Cohort Study
Source: J Clin Med. 2026 Oct 17;15(20):8063. doi: 10.3390/jcm15208063 (PMC13706473; doi:10.3390/jcm15208063)
Supplement: Supplementary file 1 [file jcm-15-08063-s001.zip › TRIPOD_AI_Checklist_R1.pdf]

Completed TRIPOD+AI Reporting Checklist – Final Revision

Clinical and Laboratory Prediction of Histopathologically Complicated Acute Appendicitis and Associated Surgical Outcomes: A Retrospective Cohort Study

Checklist basis: TRIPOD+AI (Collins GS et al., BMJ 2024;385:e078378). Official item numbering is retained; reporting requirements are concisely paraphrased.

Pagination note: Page references correspond to JCM_Manuscript_revised_R1.docx (30 rendered pages) and JCM_Supplementary_Materials_R1.docx (10 rendered pages). Section and supplementary-table references are included to remain robust to journal re-pagination.

Part A. Full TRIPOD+AI checklist

Section	Topic	Item	Applies	Reporting requirement (paraphrased)	Where reported	Completion/status
TITLE	Title	1	D;E	Identify model development/evaluation, target population, and predicted outcome.	p. 1, Title; p. 1, Abstract	Partially reported in the title; the predicted outcome is explicit, while the adult appendectomy target population is specified in the Abstract and Methods.
ABSTRACT	Abstract	2	D;E	Address the TRIPOD+AI for Abstracts reporting items.	p. 1, Abstract	Reported; see Part B of this checklist.
INTRODUCTION	Background	3a	D;E	Explain the diagnostic/prognostic context and rationale, including existing models.	pp. 1–3, Introduction	Reported: clinical context, existing scores/models, imaging considerations, and rationale are described.
INTRODUCTION	Background	3b	D;E	Describe the target population, intended purpose, position in care pathway, and intended users.	pp. 2–3, final Introduction paragraphs; pp. 26–27, Sections 4.1–4.2	Reported: intended population is adults already selected for appendectomy; nonoperative eligibility is explicitly outside the intended use.
INTRODUCTION	Background	3c	D;E	Describe relevant known health inequalities across sociodemographic groups.	pp. 8–9, Section 2.12; pp. 26–27, Sections 4.1–4.2	Known health inequalities were not characterized in the study materials. The manuscript explicitly states that fairness-specific modeling/formal sociodemographic subgroup performance analysis was not performed and should be assessed in broader cohorts.
INTRODUCTION	Objectives	4	D;E	State whether objectives involve model development, evaluation, or both.	pp. 2–3, final Introduction paragraphs	Reported: model development/internal validation, comparison with clinical scores, and later same-center temporal evaluation are stated.
METHODS	Data	5a	D;E	Describe data sources for development/evaluation, why they were used, and representativeness.	pp. 3–5, Sections 2.1 and 2.3	Reported: single-center retrospective routine-care records and the clinical data sources are described.
METHODS	Data	5b	D;E	Give dates of participant data collection/accrual and follow-up where relevant.	pp. 3–4, Section 2.1; p. 6, Section 2.7	Reported: January 2021–December 2025 accrual and 30-day postoperative outcome ascertainment are specified.
METHODS	Participants	6a	D;E	Describe setting, number of centres, and location.	pp. 3–4, Section 2.1	Reported: single-center setting at Ağrı Training and Research Hospital, Ağrı, Türkiye.
METHODS	Participants	6b	D;E	Describe participant eligibility criteria.	pp. 3–4, Section 2.1	Reported: adult appendectomy population, clinical/radiological suspicion, and definitive histopathology criteria are stated.
METHODS	Participants	6c	D;E	Report relevant treatments received and how treatment was handled in development/evaluation.	pp. 4–5, Section 2.3; p. 26, Section 4.1	Partly reported: prehospital antibiotic exposure was recorded; exact timing relative to analgesia, IV fluids, and antibiotics could not be reconstructed consistently and is acknowledged.
METHODS	Data preparation	7	D;E	Describe preprocessing and quality checking, including any relevant group-specific differences.	pp. 6–11, Sections 2.9–2.19	Reported: data completeness, fold-wise standardization, resampling, tuning, software settings, and quality-related limitations are described.
METHODS	Outcome	8a	D;E	Define predicted outcome, timing/assessment, rationale, and consistency of assessment.	pp. 5–6, Sections 2.5–2.6	Reported: primary outcome is definitive histopathological gangrenous or perforated appendicitis, with rationale and

Section	Topic	Item	Applies	Reporting requirement (paraphrased)	Where reported	Completion/status
						classification rules.
METHODS	Outcome	8b	D;E	For subjective outcomes, describe outcome assessors where available.	p. 5, Section 2.5	Reported to the extent available: classification was abstracted from final routine pathology reports; no study-specific adjudication was performed.
METHODS	Outcome	8c	D;E	Report any blinding of outcome assessment.	p. 5, Section 2.5	Reported as unavailable: pathologist blinding to clinical/intraoperative information could not be established consistently from retrospective records.
METHODS	Predictors	9a	D	Explain how initial candidate predictors were chosen and any preselection.	pp. 6–7, Section 2.9	Reported: 50 preoperative candidate predictors are listed; imaging and post-prediction variables were excluded to avoid leakage and preserve an imaging-independent model.
METHODS	Predictors	9b	D;E	Define predictors and how/when they were measured, including any blinding.	pp. 4–5 and 6–7, Sections 2.3 and 2.9	Reported: predictor definitions/list and preoperative timing are described; exact timing relative to treatment could not be reconstructed for all measurements.
METHODS	Predictors	9c	D;E	For subjectively assessed predictors, describe assessors where available.	pp. 4–5, Section 2.3; p. 26, Section 4.1	Reported: clinician-documented examination findings were not centrally reassessed; interobserver/documentation variability is acknowledged.
METHODS	Sample size	10	D;E	Explain study size and justify sufficiency; give sample-size calculation if performed.	pp. 3–4, Section 2.2	Reported: fixed retrospective cohort, no prospective sample-size calculation, 500 patients/111 events/50 candidates, and resulting model-development uncertainty are stated. A post hoc illustration using the Riley et al. criteria (approximately 1,540 patients/340 events required) is also provided.
METHODS	Missing data	11	D;E	Describe handling of missing data and reasons for omitted data.	pp. 6–7, Sections 2.8–2.9	Reported: all 50 primary candidate predictors and the outcome were complete in 500 patients; no imputation was used; other variables used available-case denominators. Complete availability was one consideration in defining the candidate set (albumin and CRP-to-albumin ratio were not entered).
METHODS	Analytical methods	12a	D	Describe how data were used for development/evaluation and any partitioning.	pp. 8–11, Sections 2.11–2.19	Reported: nested CV, repeated nested CV sensitivity analysis, bootstrap analyses, and chronological 2021–2024/2025 evaluation are described.
METHODS	Analytical methods	12b	D	Describe predictor handling: functional form, scaling, transformations, standardization.	pp. 6–10, Sections 2.9–2.14; pp. 26–27, Section 4.1	Reported: fold-wise standardization, linear continuous terms on the log-odds scale, and no splines/nonlinear transformations in the primary model. ASA class was coded as an ordinal integer; linearity was not formally assessed and is acknowledged as a limitation (Section 4.1).
METHODS	Analytical methods	12c	D	Specify model type/rationale, building steps, tuning, and internal validation.	pp. 7–10, Sections 2.10–2.14; Supplementary Tables S9–S10, pp. 8–9	Reported: L1-penalized logistic regression, tuning grid/criterion, nested validation, log-loss sensitivity analysis, and repeated nested CV.
METHODS	Analytical methods	12d	D;E	Describe handling/quantification of heterogeneity across clusters when relevant.	N/A	Not applicable: single-center study; no center-level clustering or heterogeneity analysis.
METHODS	Analytical methods	12e	D;E	Specify performance measures/plots and rationale; describe model comparisons.	pp. 8–10, Sections 2.12–2.17; pp. 16–23, Results 3.5–3.8; Figures 2–5	Reported: AUC, conditional bootstrap CI, Brier score, calibration, ROC/calibration plots, decision-curve analysis, comparator scores, and threshold metrics.
METHODS	Analytical	12f	E	Describe any model updating/recalibration arising from	N/A	No model updating or recalibration was performed after

Section	Topic	Item	Applies	Reporting requirement (paraphrased)	Where reported	Completion/status
	methods			evaluation.		evaluation; calibration was assessed but the model was not refitted to the 2025 cohort.
METHODS	Analytical methods	12g	E	Explain how predictions were calculated during model evaluation.	p. 10, Section 2.17; pp. 22–23, Section 3.8; Supplementary Table S6, pp. 5–6	Reported: the 2021–2024 model was applied unchanged to 2025, with no 2025 data used for scaling, tuning, fitting, or threshold selection; full model specification is supplied.
METHODS	Class imbalance	13	D;E	State whether imbalance methods were used and any recalibration after them.	p. 11, Section 2.19	Reported: no class weighting was used (class_weight=None); no over/under-sampling procedure is described or used.
METHODS	Fairness	14	D;E	Describe any fairness-focused analyses/methods and rationale.	pp. 8–9, Section 2.12; p. 27, Section 4.2	Reported: no fairness-specific modeling or formal subgroup performance analysis was performed because of limited single-center sample/event counts.
METHODS	Model output	15	D	Specify model output and rationale for any classification threshold.	pp. 8–9, Section 2.12; p. 10, Section 2.17; pp. 22–23, Section 3.8	Reported: model outputs individual probabilities; threshold analyses are explicitly exploratory, with a development-derived high-sensitivity threshold applied unchanged to 2025.
METHODS	Development vs evaluation	16	D;E	Identify differences between development and evaluation data in setting, eligibility, outcome, and predictors.	p. 10, Section 2.17; pp. 22–23, Section 3.8; Supplementary Table S4, pp. 4–5	Reported: development and 2025 cohorts came from the same institution with the same eligibility, outcome, predictor definitions, and data-collection framework; they differed primarily by calendar period.
METHODS	Ethics	17	D;E	Name approving ethics body and describe consent/waiver.	pp. 3–4, Section 2.1; p. 28, ethics/consent declarations	Reported: ethics committee, decision/document numbers and dates, institutional authorization, and consent waiver are stated.
OPEN SCIENCE	Funding	18a	D;E	State funding sources and funder role.	p. 28, Funding	Reported: no external funding.
OPEN SCIENCE	Conflicts of interest	18b	D;E	Disclose conflicts and financial interests.	p. 29, Conflicts of Interest	Reported: authors declare no conflicts of interest.
OPEN SCIENCE	Protocol	18c	D;E	State where protocol is accessible or that no protocol was prepared/publicly available.	pp. 3–4, Section 2.1	Reported: no prospectively registered or time-stamped statistical analysis protocol was available.
OPEN SCIENCE	Registration	18d	D;E	Provide registry information or state study was not registered.	Not reported in final manuscript	Registration status is not stated in the final source manuscript; no registry identifier or explicit “not registered” statement is provided.
OPEN SCIENCE	Data sharing	18e	D;E	Describe data availability and access restrictions.	p. 28, Data Availability Statement	Reported: data are not publicly available because of privacy/institutional restrictions; de-identified data may be available subject to approvals.
OPEN SCIENCE	Code sharing	18f	D;E	Describe availability of analytical code.	p. 11, Section 2.19; p. 28, Data Availability Statement	Reported: software/settings are described and analysis code may be available from the corresponding author subject to institutional/ethics approval.
PPI	Patient/public involvement	19	D;E	Describe patient/public involvement or explicitly state none.	Not reported in final manuscript	Patient/public involvement is not explicitly stated in the final source manuscript.
RESULTS	Participants	20a	D;E	Describe participant flow and numbers with/without outcome; follow-up where relevant.	pp. 11–12, Section 3.1 and Figure 1; pp. 14–16, postoperative outcomes	Reported for the analytical cohort and 30-day outcomes. Aggregate pre-eligibility screening counts were not retained and this limitation is acknowledged.
RESULTS	Participants	20b	D;E	Report overall characteristics, key dates/predictors/treatments, sample/event counts, follow-up, missingness, and relevant demographic differences.	pp. 11–16, Sections 3.1–3.4; Table 1; Supplementary Table S1, p. 1	Reported: participant characteristics, predictor distributions, outcome/event counts, and available-case laboratory information are presented.

Section	Topic	Item	Applies	Reporting requirement (paraphrased)	Where reported	Completion/status
RESULTS	Participants	20c	E	For evaluation, compare important predictor/demographic/outcome distributions with development data.	pp. 22–23, Section 3.8; Supplementary Table S4, pp. 4–5	Partly reported: cohort sizes and outcome prevalence are compared and the unchanged setting/definitions are stated; a full predictor-distribution comparison between periods is not tabulated.
RESULTS	Model development	21	D;E	Give participant and outcome-event counts for each development/tuning/evaluation analysis.	pp. 3–4, Section 2.2; pp. 16–21, model-development results; pp. 22–23, Section 3.8	Reported: overall n=500/111 events; temporal development n=399/90; 2025 n=101/21; analysis-specific denominators are reported where relevant.
RESULTS	Model specification	22	D	Provide the full model so predictions can be reproduced in new individuals, with access/reuse restrictions.	p. 19, final-model paragraph; Supplementary Table S6, pp. 5–6	Reported: final model intercept, 30 non-zero predictors, means/SDs, standardized and raw-scale coefficients, and probability equation are provided.
RESULTS	Model performance	23a	D;E	Report model performance with uncertainty and key subgroup performance where appropriate; use plots as helpful.	pp. 16–23, Sections 3.5–3.8; Figures 2–5; Supplementary Tables S4, S9–S10	Reported: discrimination, uncertainty, Brier score, calibration, DCA, comparator performance, threshold performance, and temporal evaluation. Formal sociodemographic subgroup performance was not undertaken.
RESULTS	Model performance	23b	D;E	Report heterogeneity across clusters if assessed.	N/A	Not applicable: single-center study with no center-level heterogeneity analysis.
RESULTS	Model updating	24	E	Report updated model and performance if model updating was performed.	N/A	No model updating/recalibration was performed after temporal evaluation.
DISCUSSION	Interpretation	25	D;E	Interpret main findings in context of objectives, prior studies, and fairness.	pp. 23–27, Discussion, including Sections 4.1–4.2	Reported: findings are interpreted against prior literature, comparator performance, same-center overprediction, fairness limitations, and clinical-use restrictions.
DISCUSSION	Limitations	26	D;E	Discuss limitations and their effect on bias, uncertainty, and generalisability.	pp. 26–27, Section 4.1	Reported extensively: retrospective single-center design, documentation/timing bias, limited events/model complexity, conditional bootstrap CI, same-center temporal evaluation, imaging limitations, fairness limitations, and surgical-selection spectrum bias.
DISCUSSION	Usability in current care	27a	D	Explain how poor-quality or unavailable predictor inputs would be handled at implementation.	p. 27, Section 4.2	Reported: because the model is not externally validated/implemented, no validated strategy for unavailable predictor values has been specified.
DISCUSSION	Usability in current care	27b	D	State required user interaction/expertise for model use.	p. 27, Section 4.2	Reported: no clinical user interface/workflow has been developed; user-interaction requirements are deferred to external validation and prospective implementation testing.
DISCUSSION	Usability in current care	27c	D;E	Describe next research steps focused on applicability and generalisability.	p. 27, Section 4.2; pp. 27–28, Conclusions	Reported: independent multicenter validation, calibration/recalibration assessment, prospective decision-impact evaluation, and imaging-inclusive comparisons are identified as next steps.

Part B. TRIPOD+AI for Abstracts checklist

Section	Item	Requirement (paraphrased)	Where reported	Status
Title	1	Identify the study as a prediction-model development/evaluation study, target population and outcome.	p. 1, Title; p. 1, Abstract	Partially reported in the title; target population is explicit in the Abstract/Methods.
Background	2	Briefly state clinical context and rationale.	p. 1, Abstract	Reported.

Section	Item	Requirement (paraphrased)	Where reported	Status
Objectives	3	State whether model development, evaluation, or both are undertaken.	p. 1, Abstract	Reported: development/internal validation and same-center temporal evaluation are described.
Methods	4	State data source/design.	p. 1, Abstract	Reported: single-center retrospective cohort.
Methods	5	State eligibility and setting.	p. 1, Abstract	Reported: 500 adults undergoing appendectomy for suspected acute appendicitis.
Methods	6	Define the predicted outcome.	p. 1, Abstract	Reported: gangrenous or perforated appendicitis on histopathology.
Methods	7	Summarize model type, building steps and internal validation.	p. 1, Abstract	Reported: LASSO logistic regression with nested stratified cross-validation, bootstrap predictor-selection reproducibility assessment, calibration, and same-center temporal evaluation.
Methods	8	State performance measures.	p. 1, Abstract	Reported: AUC, Brier score, calibration, and comparator-score performance.
Results	9	Report sample size and outcome events.	p. 1, Abstract	Reported: 500 participants and 111 primary-outcome events.
Results	10	Summarize final-model predictors.	p. 1, Abstract; Supplementary Table S6, pp. 5–6	Partly reported in the abstract: the retained predictor set is not enumerated; the complete final model is supplied in Supplementary Table S6.
Results	11	Report model performance with confidence intervals.	p. 1, Abstract	Reported: primary AUC with CI, Brier/calibration, AIR/Alvarado comparisons, and same-center 2025 AUC are included.
Discussion	12	Provide balanced overall interpretation.	p. 1, Abstract conclusion	Reported: no significant advantage over AIR, same-center overprediction, and need for independent multicenter validation/recalibration are stated.
Registration	13	Give registration details, or state not registered.	Not reported in Abstract or final manuscript	Registration status is not stated in the supplied final manuscript.

Final audit note: Items 18d (study registration) and 19 (patient/public involvement) are marked as not reported because the supplied final manuscript does not contain explicit statements on these points. No unsupported assumptions were added to the checklist.

Reference

Collins GS, Moons KGM, Dhiman P, et al. TRIPOD+AI statement: updated guidance for reporting clinical prediction models that use regression or machine learning methods. BMJ. 2024;385:e078378. doi:10.1136/bmj-2023-078378.
